# Local unemployment changes the springboard effect of low pay: Evidence from England

**DOI:** 10.1371/journal.pone.0224290

**Published:** 2019-11-13

**Authors:** Alexander Plum, Gundi Knies

**Affiliations:** 1 New Zealand Work Research Institute, Auckland University of Technology, Auckland, New Zealand; 2 Institute for Social and Economic Research, University of Essex, Colchester, England, United Kingdom; TED University, TURKEY

## Abstract

There is considerable debate on whether the employment and earnings prospects are better for those on low pay or for the unemployed. Low-pay work tends to be undertaken more locally but no empirical analysis has focused on how local opportunities alter prospects. Using Understanding Society data for England matched with local unemployment rates, we estimate dynamic random effects panel models, which show robust evidence that the future unemployment risk is lower for those who are currently on low pay compared to those who are currently unemployed. The low-paid also have a higher chance than the unemployed of becoming higher-paid. These findings are most marked in neighbourhoods with high unemployment.

## Introduction

Whilst unemployment is an undesirable position for an individual to be in, the jury is still out on whether any kind of employment is preferable to unemployment (see, e.g., [1]). Many countries, the UK in particular, have seen the establishment of a low wage sector which, so the common assertion goes, offers ‘bad’ jobs that involve part-time, non-permanent and short-term employment contracts, and that do not provide access to training or transferable skills (see, e.g., [2], [3]). In this view, low-pay employment has little positive impact on the worker’s human capital stock and may not provide the worker with access to more promising employment networks. On the other hand, being low-pay employed may nevertheless signal a lesser human capital depreciation than being unemployed, and it may signal the willingness to work, both of which should translate into positive labour market outcomes such as a lower risk of becoming unemployed and entering higher-pay employment more easily, compared to unemployment. Such positive returns to signalling have been shown, for instance, in the literature on unpaid overtime and may be higher in areas with high local unemployment (see, e.g., [4]).

There is, in fact, a long and rich tradition in the theoretical literature devoted to linking individuals′ labour market outcomes and opportunities to the local context. Galster [5] lists 15 potential causal pathways through which the neighbourhood context may impact decision-making. Pathways include peer effects in the accumulation of human capital (see, e.g., [6]), harmonization of work attitudes (see, e.g., [7]), use of social networks as an informal job market—which has been suggested to be particularly relevant for low-skilled workers ([8], [9])—and spatial discrimination by the employer (see, e.g., [10]). It has also been demonstrated that differences in the local unemployment rate are sizeable and persistent (see, e.g., [11]), affecting, e.g., the probability of exiting unemployment ([12], [13], [14]).

Many Western countries face a persistently high share of low-pay employment ([15]). One explanation for this phenomenon is the rise of the ‘gig economy’ as a major contributor to the creation of new jobs (e.g., [16]). The so-called sharing economy operates in various fields that “span diverse sectors and industries and offer a wide variety of goods and services including accommodation (e.g. AirBnB), transportation (e.g. Zipcar, Lyft, Hailo, and Uber), finance (e.g. Kickstarter), recycling (e.g. Freecycle), and ways to organize and prioritize tasks (e.g. Taskrabbit)” ([17], p. 2). In recent years, working conditions and earnings in these economies have come under scrutiny (e.g., [18]) for fostering precarious employment and undercutting worker’s rights (e.g., [19]). Many of the jobs in these economies are outside the traditional employer and employee relationship, for example, because workers are treated as self-employed.

The aim of this study is to advance the empirical research on low-pay dynamics by using longitudinal data for England linked with local labour market information at the level of very immediate neighbourhoods. Whilst we may expect that the prospects of advancing one′s career declines as competition for jobs in the local labour market gets fiercer -simply as the number of available higher-pay jobs is lower and the number of applicants is higher- given the expansion of low-pay and ‘bad’ jobs in many areas, we ask whether the unemployed and low-pay employed suffer equally from living in a neighbourhood that has a high level of unemployment, or whether the effects are related to the individual labour market position. We hypothesize that low-pay employment will be more advantageous in local areas with high unemployment. This would be in line with the conjecture that low-pay workers signal to employers their willingness to work and that they are actively counteracting the deterioration of their human capital; a signal which employers use to screen job applications.

Whilst the emerging empirical research on low-pay dynamics has produced evidence both in support and in contradiction to the hypothesis that low-pay employment is a steppingstone to higher-pay employment and that it is, therefore, preferable to unemployment (see, e.g., [20] and [21]) our study is the first in the field of low-pay dynamics to take into account that employment trajectories may also depend on where one lives (and that unemployment may prompt people to move [22]). More specifically, this research contributes to the empirical research on the steppingstone effect of low pay by taking into account heterogeneity in local labour market conditions and examining whether local competition for jobs alters the prospects of higher-pay employment and the risk of experiencing unemployment.

There are a number of empirical challenges not only to estimating dynamic panel models but also to identifying neighbourhood effects (see, e.g., [23], [24]) and we will address these inasmuch as is possible. In particular, to address common specification issues in dynamic modelling, we will include a random effects error term to address the problem that people may differ in their unobservable characteristics ([25]), which may be correlated between the mutually exclusive labour market positions (see, e.g., [21]), and we consider that the initial labour market positions may not be randomly assigned ([26]).

With respect to issues related to identifying neighbourhood effects, we test labour market characteristics at three geographical scales, ranging from the very immediate neighbourhood to the regional level. Whilst we believe that the signalling and “employer screening” effect will operate at the very local level testing the effects at greater scales provides indirect assurance that this specific effect is not also operating at greater scales. Among our robustness tests we, moreover, restrict the sample to non-movers which allows us to isolate the effects of unobserved neighbourhood characteristics (as suggested by [27] and [5]), and we use the neighbourhood indicator at a larger scale to alleviate the concern that people may have chosen the particular neighbourhood for the employment prospects it offers (or, put another way, that the labour market outcomes and the neighbourhood profiles are endogenous). We will not attempt to model selection into neighbourhoods and neighbourhood change directly; this would require even more powerful data than we have access to, i.e., with a greater number of movers across different types of neighbourhoods as choosing to remain in a changing or even declining neighbourhood is a form of selection that can be just as consequential as the decision to relocate.

We draw on data from Understanding Society, a very large nationally representative panel survey for the UK, matched with local labour market indicators for England. These indicators of labour market characteristics at the level of neighbourhoods have the advantage of capturing more appropriately any heterogeneity in the labour market affecting those in the low wage sector than has been possible in previous analyses, e.g., when using regional indicators (see, e.g., [20]): Work at low pay tends to be undertaken locally—the average distance to work for those on low pay was 5.6 miles in 2010/2011 whilst it amounted to 11.8 miles for those on higher-pay (population estimates based on own analysis of Understanding Society (2015), Wave 2. The low pay threshold set at £7.48 gross hourly wage and the appropriate population weights were applied). This means that the unemployment rate in the immediate neighbourhood may be a better proxy for the applicant pool from which the employer may cherry-pick than if the rate were computed at a larger geographical scale.

The results indicate that there is a clear negative association between the local unemployment rate and employment and income prospects, which is affecting the long-term unemployed more than the short-term unemployed than the low-paid and the higher-paid. With respect to the steppingstone effect of low pay, irrespective of the local unemployment rate, the long-term unemployed can profit considerably from taking up low-pay employment as not only the risk of becoming unemployed is lowered but the chance of becoming higher-paid employed in the subsequent period is increased. By contrast, there is little difference in the prospects for the short-term unemployed and the low-paid, except in neighbourhoods with the highest levels of unemployment where the low-paid fare significantly better.

This finding is in line with the conjecture that human capital deteriorates during an unemployment spell and that the likelihood of entering employment is lower the longer unemployment has commenced. It also emphasizes the urgency to signal the willingness to work when competition for jobs in the local area is high. Overall, the results suggest that entering low-pay employment is preferable—in terms of lowering the risk of future unemployment and increasing the chances to enter higher-pay employment—to unemployment, especially when local unemployment is high. These results are robust to a range of sensitivity tests.

## Literature review

### Unemployment persistency

There are a number of theoretical contributions that incorporate the experience of unemployment in labour market models. An early example is provided by Vishwanath [28], who posited that unemployment sends a negative signal as firms view the unemployment duration as an indicator for the productivity level. This stigma, when considered in a job search model, means that the risk of staying unemployed increases with the unemployment duration, also referred to in the economic literature as the negative duration dependence in unemployment. Blanchard and Diamond [29] examined in a labour market model with job creation, destruction and matching the effect of incorporating the length of unemployment in a firm′s hiring decision on the exit rates out of unemployment. The authors demonstrated that if the applicants′ unemployment duration is chosen by the firm as a ranking device in the hiring process, the exit probability of employed workers, were they to become unemployed, is higher than that of an already unemployed worker. Moreover, in labour markets with a low unemployment rate, applicants face less competition for vacancies, and with an increasing level of unemployed, duration dependence in unemployment increases.

A further theoretical explanation for unemployment persistence has been presented by Acemoglu [30]. Under the assumption that the unemployed face a deterioration of their skills during a spell of unemployment and that maintaining the skill level is both costly and not observable, firms will discriminate against the unemployed. In response to this discrimination, no measures will be undertaken by the unemployed to improve their level of skills. In the equilibrium, this will result in some negative duration dependence in unemployment, seeing as the exit probability declines with the length of the unemployment spell. Note, however, that the high-skilled unemployed may be willing to wait for an appropriate job offer (i.e., high-quality jobs), hence, increase the duration of their unemployment voluntarily. Pissarides [31] has suggested that this preference to wait for higher quality jobs is being considered in the employers′ hiring decisions. In other words, the high-skilled will not necessarily suffer from a scarring effect in unemployed.

Empirical evidence for the scarring effect of unemployment has been presented for many countries and using both survey and experimental data. Based on survey data, for the US, little evidence for the scarring effects is found (see, e.g., [32]), whereas strong evidence is found for the UK ([33]), Germany ([34]), Australia ([35]), Spain ([36]), Norway ([37]) and Europe ([38]). Empirical evidence for the duration dependence in unemployment has also been presented on the basis of experimental data for Switzerland ([39]) and the US ([40]), but little evidence for stigma effects of unemployment was found in Swedish experimental data (except for the longer-term unemployed, see [41]). Moreover, a number of studies have shown that duration dependence in unemployment is more pronounced in markets with low unemployment ([40], [42], [43]).

### Employment and earnings prospects for low-pay workers

While there is broad consensus in the theoretical literature that there exists negative duration dependence in unemployment, theoretical predictions about the direction of the effect of low pay on employment and earnings prospects are less clear. On the one hand, taking up employment will attenuate, if not stop, the deterioration of human capital. In addition, workers signal their willingness to work even if the pay is low. On the other hand, the type of job might reveal some below average productivity. McCormick [44] has shown that skilled workers tend to avoid unskilled jobs, as skilled jobs are more satisfying to them. When becoming unemployed, the high-skilled will spent their time searching for an adequate job and will not take up poorly paid employment in the interim, and firms use the respective search strategy of the unemployed as a screening device for productivity. This mechanism led to Layard’s [1] famous remark that “while unemployment is a bad signal, being in a low-quality job may well be a worse one.” [p. 249].

Given these counteracting forces, it is perhaps little surprising that empirical results on the employment prospects of low-pay workers are heterogeneous. It has been suggested that five percent of the UK’s workforce were in persistently low pay in the first decade of the 2000s ([45]). Using data from the British Household Panel Survey (BHPS) and applying a range of random-effects and fixed-effects estimators, Steward [20] found no statistically significant differences between low-pay workers and the unemployed in their employment prospects (expressed as the likelihood of entering unemployment). However, Plum [21] presents indications that especially for long-term unemployed low-wages might provide a gateway to higher wages in the future. Low-pay workers have also been shown to have better chances than the unemployed to climb up the salary ladder in Germany ([46], [47], [48],[49]) and Australia ([50]). However, for Italy, Cappellari [51] found a high persistence in low pay and the author concludes that the accumulation of human capital has only a minute impact on exiting the low-wage sector. Moreover, high persistence in low pay has been documented for many countries in Europe [52].

### The impact of local labour market conditions

Several theoretical contributions suggest how the neighbourhood context influences individuals’ labour market position and how individual behaviour leads to neighbourhood inequality in the aggregate. Galster and Killen [53] provide a general perspective on how the local context influences young peoples’ prospective socioeconomic status. In their model, decision-making depends upon actual and perceived opportunities. Opportunities and how they are perceived are influenced, on the one hand, by malleable and unmalleable personal characteristics (such as age, gender and family background and past decisions), and on the other hand by objective local circumstances (e.g., existence of institutions, labour market, housing), by the local social network (e.g., family, friends, neighbours), and by the characteristics of the neighbours (e.g., quality of schools). These shape individual values, aspirations and preferences but also define the local opportunity structure. More specific models for how the neighbourhood context affects decisions include that by Akerlof [54] who shows that the local social code to not work for an unfair wage can result in voluntary unemployment, and that by Streufert [55] who shows that the absence of high-income earners as positive role models from the neighbourhood can depress schooling because the distribution of returns to education that is observable to teenagers in the deprived neighbourhood is skewed, and not representing the more favourable national distribution of returns to education.

A plethora of empirical research has shown that neighbourhood characteristics have an impact on individual labour market outcomes, for instance, the higher the local unemployment rate the longer people spent receiving income maintenance support (see, e.g., [12]), the less likely they are to transition from welfare to work (see, e.g., [13]), or, more generally, from unemployment into work (see, e.g., [14]). In the context of unemployment and low-pay dynamics, the focus has been on rather rough headline indicators such as the regional unemployment rate (see, e.g., [23]) which may not be the most appropriate scale at which some of the hypothesized neighbourhood effects may operate (for a discussion see [24]). In particular, we would expect that the availability and accessibility of (good) jobs in the local labour market influences labour market trajectories such as unemployment persistency or climbing up the pay distribution as those with scarce economic resources will tend to look for local jobs, and not, for example, for jobs in the entire travel to work area (used, e.g., by [20]), which divide the whole of UK into just 228 areas and treat the entire area of Greater London as one spatial unit. To our knowledge, no study has reported whether the local level of unemployment (or indeed any other local labour market indicator) affects the prospects of entering higher-pay employment and whether there are advantages to taking on low-pay employment in the first instance.

The aim of this study, then, is to advance the empirical research on low-pay dynamics by using longitudinal data for England linked with local labour market information at the level of very immediate neighbourhoods. Whilst we may expect that the prospect of advancing one′s career declines as competition for jobs in the local labour market gets fiercer -simply as the number of available higher-pay jobs is lower and the number of job applicants is higher- given the expansion of low-pay and ‘bad’ jobs in many local areas, we ask whether the (short- and long-term) unemployed and low-pay employed suffer equally from living in a neighbourhood that has a high level of unemployment, or whether the effects are specific to the labour market position. We hypothesize that the steppingstone effect of low-pay employment will be more marked in neighbourhoods with high unemployment. This effect would be in line with the conjecture that low-pay workers signal to employers their willingness to work and they actively counteract the deterioration of their human capital.

## Data and descriptive statistics

### Understanding Society

We use data from the first five waves of Understanding Society, the UK Household Longitudinal Study ([56]). The study started in 2009 with around 26,000 private households, which were randomly selected to participate using a clustered and stratified sample design. Since the second wave, which took place in 2010/11, the Study also incorporates around 8,000 households who were previously interviewed as part of the British Household Panel Survey (BHPS). Interviews are conducted annually with interviewers calling at the respondents′ home and attempting to interview all adults (aged 16 years or older) living in responding households. The study collects a wealth of information relating to the respondents’ economic and social circumstances, their values and attitudes, and provides a detailed picture about how people move into and out of employment, how their pay and other life circumstances change. The study design and content closely follow the basic design of other longitudinal household panel studies, which have been employed to investigate employment transitions, such as the aforementioned BHPS, the American Panel Study of Income Dynamics (PSID) and the German Socio-economic Panel (SOEP). For more detailed information about Understanding Society, see [57].

Understanding Society is particularly well suited for the analysis. In addition to providing the relevant individual characteristics, the survey design assured that the sample is nationally representative for all Government Office Regions of the United Kingdom and that there are enough respondents from metropolitan, urban and rural areas within them to provide enough statistical power for results on local area influences on individual outcomes. Moreover, it is possible to access look-up files between the respondent′s home address and official geographical identifiers at very immediate scales. This allows us to augment the data from the Study with published time series data on labour market indicators for England at those geographical scales.

The focus of analysis necessitated our sample to be restricted to respondents who, in at least four consecutive survey waves, were either employed (full-time or part-time) and reporting some positive number of hours worked in a current job and a positive amount of gross pay, or self-define as unemployed. For parsimony, we restricted the sample to males aged 25–55 years living in England. The sample is restricted to England because comparable local labour market indicators are not available for other parts of the UK. Females, the self-employed, full-time students, retirees and those who are long term sick or disabled–groups who dominate the low pay sector—have also been excluded as their labour market transitions are likely to follow different patterns which to explore is beyond the scope of this analysis of neighbourhood context effects on low pay dynamics.

Our key variable of interest is a marker of respondent′s employment status, which can assume three states: unemployed, low-pay employed, and higher-pay employed. Following the standard definition by the Organization of Economic Cooperation and Development (OECD), a pay is considered low if the gross hourly wage is below two-thirds of the respective annual median gross hourly wage, and higher otherwise ([58]). Table 1 reports the respective annual low-pay thresholds for England over the study period. It can be seen that the threshold increased somewhat, starting at £6.92 in 2009 and increasing up to £8.31 in 2013.

**Table 1:** Low-pay thresholds.

Year	2009	2010	2011	2012	2013
Threshold in £	6.92	7.48	7.90	8.00	8.31

*Source*: Understanding Society (2015), Waves 1–5, 2009–2014. Based on the weighted gross hourly wages of both males and females in England.

To allow for effect heterogeneity for the long-term and short-term unemployed (see, e.g., [20]), those who were unemployed in *t-1* and had no employment spell since the last interview in *t-2* are considered long-term unemployed, and those who had some employment spell since their interview in *t*-2 are considered short-term unemployed. Unemployed new entrants to the study are defined as long-term unemployed if they were not employed in the last 12 months before the interview, and short-term unemployed otherwise.

Employing these sample restrictions and variable definitions, the final balanced sample consists of 2,477 respondents who were observed over the entire four year period (yielding 8,738 person-year observations); the sample spent 77.1per cent of the time in high pay, 18.0per cent in low pay, 2.0per cent in short- and 2.8per cent in long-term unemployment. Table 2 shows that there was considerable movement into and out of these positions.

**Table 2:** Persistence and change in labour market positions (transition matrix).

	Higher-Paid_t_	Low-Paid_t_	Unemployed_t_
Higher-Paid_t-1_	93.82	4.67	1.51
Low-Paid_t-1_	21.00	74.92	4.08
Short-term unemployed_t-1_	36.20	31.90	31.90
Long-term unemployed_t-1_	4.84	12.90	82.26
Total_t_	77.16	18.01	4.83

*Source*: Understanding Society (2015), Waves 1–5, 2009–2014. *N* = 8,738.

### Neighbourhood data

We obtained permission to access a look-up file between household identifiers and Lower Super Output Area (LSOA) codes to allow us to augment the panel data with relevant neighbourhood context information from published tables using that identifier. LSOA are used to monitor regeneration in England and comprise of between four and six census output areas (OAs), covering ~ 600 households with ~ 1,500 people on average. Households are grouped together based on spatial proximity, natural boundaries and homogeneity of dwelling type and tenure. LSOAs are “substantially smaller and more internally homogenous than the area geographies that have been relied upon by many previous (area) studies, enhancing our ability to uncover evidence of neighbourhood processes operating within local communities” [59], p. 1055–1056). There were 32,482 LSOA in England in 2001, and the panel study has members in around one-third of them. On the basis of look-up files between LSOA and greater geographical scales, it was also possible to construct labour market indicators at less immediate scales for robustness tests. In particular, we use the official geographical units of Local Authority Districts and Government Office Regions (which are also known as LAU1 and NUTS1, respectively, statistical units in Europe).

### Neighbourhood unemployment

Official unemployment rates in the UK are measured using two alternative approaches (see, e.g., [60]). The first approach uses the ILO definition, described above, and the second measure is the ratio of claimants of Job Seeker’s Allowance (i.e., an income maintenance payment to those out of work actively seeking employment) to the economically active population aged 16–74. The ILO definition is preferred in labour market research, but it is not available at the small neighbourhood scales we are interested in so we have to use the claimant count measure. At the local authority level (the lowest geographical scale for which national statistics report the ILO measure), the two rates are very highly correlated (i.e., greater 0.9 in the years under focus here). There were also no statistically significant differences in the empirical results when we swapped the rates at this higher level of spatial aggregation.

We sourced the claimant count and population estimates at the LSOA level from the Department for Transport (DfT) Accessibility Statistics 2013. The statistics provide information about access to eight domains of public services in the immediate areas in which study members live and have been linked with Understanding Society ([61]). Accessibility data in the employment domain allow us to calculate the local unemployment rate as the proportion of claimants of Job Seeker’s Allowance (JSA) (i.e., “users at risk (of being excluded from employment)” in Accessibility Statistics terminology) to the economically active population aged 16–74 (i.e., “users of employment centres” in Accessibility Statistics terminology).

**Fig 1:**
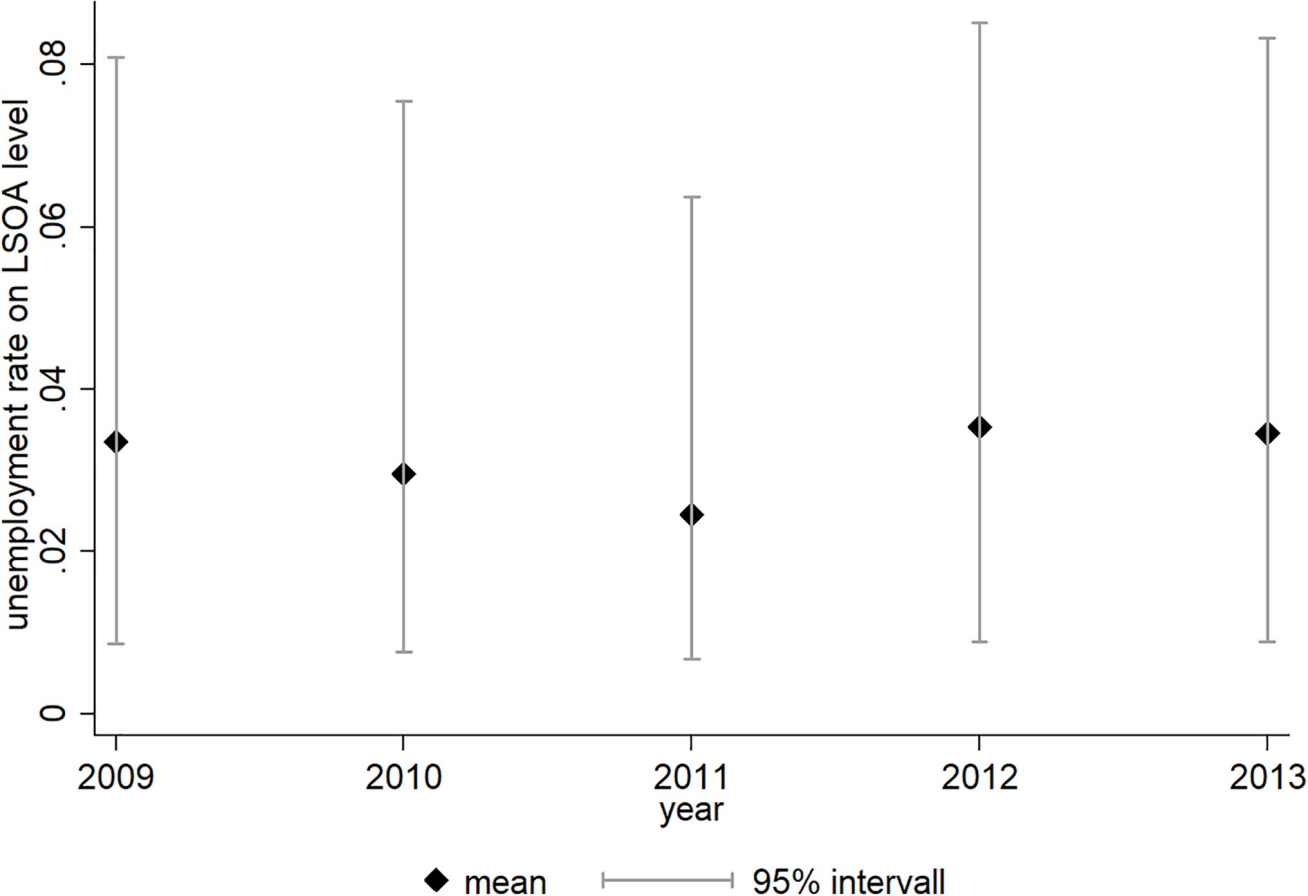
Annual distribution of the unemployment rate at LSOA-level. *Source*: DfT Accessibility Statistics 2013. The figure shows the annual distribution of the unemployment rate on LSOA level.

Fig 1 shows that there is some variation in neighbourhood unemployment rates thus defined, both within and across years. With correlation coefficients ranging from 0.895 to 0.982, the relative position of LSOAs within the neighbourhood unemployment distribution remains fairly constant over time (see S1 Table in the Supplemental Material).

**Fig 2:**
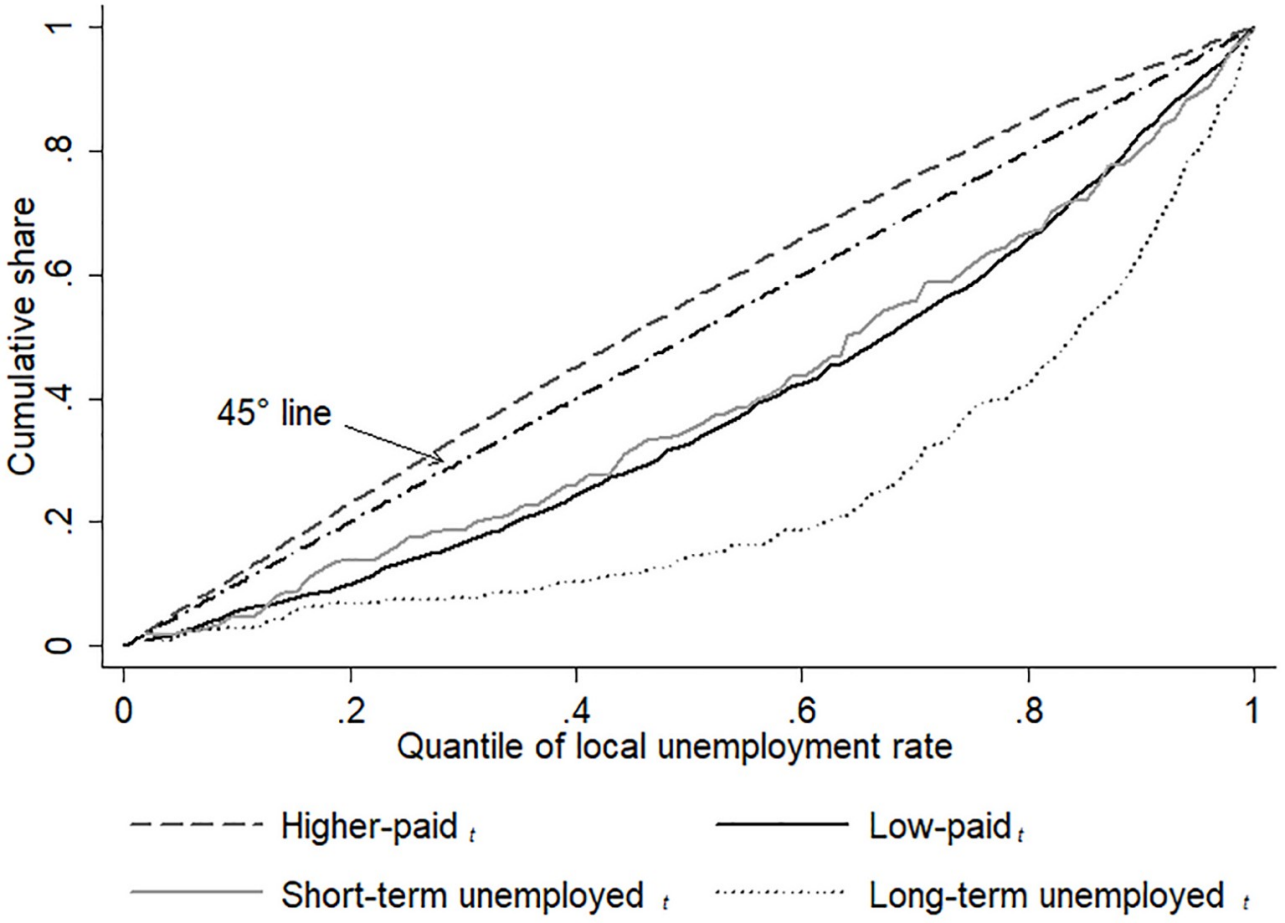
Distribution of labour market positions in *t*. *Source*: Understanding Society (2015), Waves 1–5, 2009–2014; linked with DfT Accessibility Statistics 2013.

To illustrate the distribution of labour market positions in relation to local labour market tightness, Fig 2 presents for each labour market position the cumulative share with respect to the local unemployment level (see also S2 Table). It can be seen that depressed labour markets are characterized by a triad of large shares of long- and short-term unemployed and of workers on low pay: about 60 per cent of the long-term unemployed live in neighbourhoods that belong to the two quantiles with the highest unemployment rates and about 60 per cent of the short-term unemployed and the low-paid employed belong to the four quantiles with the highest unemployment rates.

We also include an LSOA-level indicator of urbanity to consider heterogeneity in other structural factors that impact employment and earnings prospects and may be correlated with the local unemployment rate. The indicator is taken from the DfT National Travel Survey (NTS) and classifies each address in 2001 into one of four metropolitan areas, one of six urban areas with a population of at least 100,000 inhabitants, or rural areas. Note that in contrast to regions or local authority boundaries, the urbanity marker considers the spatial (dis)connectedness of build-up areas to decide which area type is appropriate. This means, for instance, that not all addresses in the region London are also classified as falling into the NTS area type “Inner or outer London metropolitan area” as this depends on whether or not it is connected to the bulk of the metropolitan area addresses.

Descriptions of all variables used in the econometric models and basic descriptive statistics are reported in and S4 Table in the Supplementary Materials, respectively.

### Econometric model

#### The baseline model

The aim of this study is to analyse the effect of the previous labour market position on employment and earnings prospects. An appropriate statistical model for this complex problem is a correlated dynamic random-effects probit model, as it has been suggested by Steward [20] and as it also has been applied in a low-pay study by Knabe and Plum [44].

The statistical representation of the model is as follows (see Steward [20]). The observed binary outcome variables are defined as:

$$y_{it}^{\left(\text{ue}\right)}=\left\{ \begin{array}{cc} 1 & \text{if}\;\text{the}\;\text{person}\;\text{is}\;\text{unemployed,}\\ 0 & \text{otherwise,} \end{array}\right.$$ (1a)

and,

$$y_{it}^{\left(\text{hp}\right)}=\left\{ \begin{array}{cc} 1 & \text{if}\;\text{the}\;\text{person}\;\text{is}\;\text{higher-paid}\;\text{employed,}\\ 0 & \text{otherwise}, \end{array}\right.$$ (1b)

and,

$$y_{it}^{\left(\text{lp}\right)}=\left\{ \begin{array}{cc} 1 & \text{if}\;\text{the}\;\text{person}\;\text{is}\;\text{low-paid}\;\text{employed,}\\ 0 & \text{otherwise}. \end{array}\right.$$ (1c)

Note that the labour market positions are mutually exclusive; each observation can only be in one of the three labour market positions. Note that we do not observe transitions from higher-paid or low-paid employment into long-term unemployment. This is because we cannot differentiate the length of unemployment for those currently unemployed as per our definition the long-term unemployed have to be unemployed at two consecutive interviews. The model is defined as:

$$y_{it}^{\left(\text{ue}\right)}=1\left\{y_{i(t-1)}^{\left(\text{hp}\right)}\gamma_{11}+y_{i(t-1)}^{\left(\text{lp}\right)}\gamma_{12}+y_{i(t-1)}^{\left(\text{ue-short}\right)}\gamma_{13}+{\text{ue-rate}}_{i(t-1)}\delta_{1}+x_{it}^{\prime }\beta_{1}+\kappa_{1i}+u_{1it}>0\right\}$$ (2a)

and if $y_{it}^{\left(ue\right)}=0$,

$$y_{it}^{\left(\text{hp}\right)}=1\left\{y_{i(t-1)}^{\left(\text{hp}\right)}\gamma_{21}+y_{i(t-1)}^{\left(\text{lp}\right)}\gamma_{22}+y_{i(t-1)}^{\left(\text{ue-short}\right)}\gamma_{23}+{\text{ue-rate}}_{i(t-1)}\delta_{2}+x_{it}^{\prime }\beta_{2}+\kappa_{2i}+u_{2it}>0\right\}$$ (2b)

The variable $y_{it}^{\left(\text{ue}\right)}$ (and $y_{it}^{\left(\text{hp}\right)}$, respectively) refers to the labour market position of individual *i* = 1, …, *N* at time *t* = 1, …, *T*. Furthermore, explanatory variables (*x*_*it*_) influence the labour market position (see S3 Table). Moreover, it is assumed that the labour market position in the previous period (i.e., $y_{i(t-1)}^{\left(\text{hp}\right)}$, $y_{i(t-1)}^{\left(\text{lp}\right)}$ and $y_{i(t-1)}^{\left(\text{ue-short}\right)}$, respectively) has an impact on the current position. Note that being long-term unemployed is chosen as the reference category. To capture the effect of the unemployment level in the neighbourhood, the continuous unemployment marker on the LSOA level (ue-rate_*i*(*t*−1)_) is included. The lagged neighbourhood indicator is used to avoid interrelation of the current labour market position with the current conditions in the neighbourhood. For movers, the local conditions of the destination neighbourhood are used (in a robustness estimation, we controlled for the size of the LSOA by including the population aged 16–74 as an explanatory variable; the results remained unchanged). As noted by Heckman [25], individuals may not only differ in observable but also in unobservable characteristics; therefore, individual-specific time-constant error terms *k*_*ji*_ with *j* ∈ {1,2} are included. The time-specific idiosyncratic error term is denoted *u*_*jit*_.

However, it is likely that the labour market position in the initial period is not randomly distributed and in fact correlated with the random-effects, referred to in the literature as the initial conditions problem [26]. To address the initial conditions problem, we follow Wooldridge’s [62]’s suggestion, i.e., we condition the dynamic sequence of the estimation on the outcome in the initial period. Referring to the individual-specific time-invariant error term of Eqs (2a) and (2b), *κ*_*ji*_ takes the following form (see [58]):

$$\kappa_{ji}=a_{0j}+y_{i0}^{\left(\text{hp}\right)}\tau_{j1}+y_{i0}^{\left(\text{lp}\right)}\tau_{j2}+y_{i0}^{\left(\text{ue-short}\right)}\tau_{j3}+{\overset{-}{x}}_{i}\pi_{j}+z_{i}y_{i0}^{\left(\text{hp}\right)}\eta_{j1}+z_{i}y_{i0}^{\left(\text{lp}\right)}\eta_{j2}+z_{i}y_{i0}^{\left(\text{ue-short}\right)}\eta_{j3}+\alpha_{ji}$$ (3)

Plugging Eq (3) into Eqs (2a) and (2b) leads to:

$$y_{it}^{\left(\text{ue}\right)}=1\left\{y_{i(t-1)}^{\left(\text{hp}\right)}\gamma_{11}+y_{i(t-1)}^{\left(\text{lp}\right)}\gamma_{12}+y_{i(t-1)}^{\left(\text{ue-short}\right)}\gamma_{13}+{\text{ue-rate}}_{i(t-1)}\delta_{1}+x_{it}^{\prime }\beta_{1}+y_{i0}^{\left(\text{hp}\right)}\tau_{11}+y_{i0}^{\left(\text{lp}\right)}\tau_{12}+y_{i0}^{\left(\text{ue-short}\right)}\tau_{13}+{\overset{-}{x}}_{i}\pi_{1}+z_{i}y_{i0}^{\left(\text{hp}\right)}\eta_{11}+z_{i}y_{i0}^{\left(\text{lp}\right)}\eta_{12}+z_{i}y_{i0}^{\left(\text{ue-short}\right)}\eta_{13}+\alpha_{1i}+u_{1it}>0\right\}$$ (4a)

and if $y_{it}^{\left(\text{ue}\right)}=0$,

$$y_{it}^{\left(\text{hp}\right)}=1\{y_{i(t-1)}^{\left(\text{hp}\right)}\gamma_{21}+y_{i(t-1)}^{\left(\text{lp}\right)}\gamma_{22}+y_{i(t-1)}^{\left(\text{ue-short}\right)}\gamma_{23}+{\text{ue-rate}}_{i(t-1)}\delta_{2}+x_{it}^{\prime }\beta_{2}+y_{i0}^{\left(\text{hp}\right)}\tau_{21}+y_{i0}^{\left(\text{lp}\right)}\tau_{22}+y_{i0}^{\left(\text{ue-short}\right)}\tau_{23}+{\overset{-}{x}}_{i}\pi_{2}+z_{i}y_{i0}^{\left(\text{hp}\right)}\eta_{21}+z_{i}y_{i0}^{\left(\text{lp}\right)}\eta_{22}+z_{i}y_{i0}^{\left(\text{ue-short}\right)}\eta_{23}+\alpha_{2i}+u_{2it}>0\}$$ (4b)

As normalizations for the random-effects error terms, it is assumed that $\alpha_{ji}\sim N(0,\sigma_{\alpha_{j}}^{2})$ and the two random-effects may be correlated with the correlation parameter *ρ*_*α*_. For identification, it is assumed that the idiosyncratic error terms are standard-normal distributed, i.e., *u*_*jit*_ ~ *N*(0,1). Note that the composite error term is *υ*_*jit*_ = *α*_*ji*_ + *u*_*jit*_ and is correlated over time in the following way $\lambda_{j}=corr(\upsilon_{jit},\upsilon_{jis})=\frac{\sigma_{\alpha_{j}}^{2}}{\sigma_{\alpha_{j}}^{2}+\sigma_{u_{j}}^{2}}$ for each *t* ≠ *s*. The variance-covariance matrix of the random-effects error terms, then, takes the following form:

$$V_{\alpha }=(\begin{array}{cc} \sigma_{\alpha_{1}}^{2} & .\\ \rho_{\alpha }\sigma_{\alpha_{1}}\sigma_{\alpha_{2}} & \sigma_{\alpha_{2}}^{2} \end{array})$$ (5)

Furthermore, it may be that the idiosyncratic shocks are correlated between both processes. The individual outcome probabilities are:

$$P_{it}(\alpha_{1}^{*},\alpha_{2}^{*})={{\left\{\Phi [\mu_{1}\right]\}}^{y_{it}^{\left(\text{ue}\right)}}\left\{\Phi [-\mu_{1}]\Phi [(2y_{it}^{\left(\text{hp}\right)}-1)\mu_{2}\right]\}}^{\left(1-y_{it}^{\left(\text{ue}\right)}\right\}}$$ (6)

and Φ refers to the cumulative univariate normal distribution function and

$$\mu_{j}=y_{i(t-1)}^{\left(\text{hp}\right)}\gamma_{j1}+y_{i(t-1)}^{\left(\text{lp}\right)}\gamma_{j2}+y_{i(t-1)}^{\left(\text{ue-short}\right)}\gamma_{j3}+{\text{ue-rate}}_{i(t-1)}\delta_{j}+x_{it}^{\prime }\beta_{j}+y_{i0}^{\left(\text{hp}\right)}\tau_{j1}+y_{i0}^{\left(\text{lp}\right)}\tau_{j2}+y_{i0}^{\left(\text{ue-short}\right)}\tau_{j3}+{\overset{-}{x}}_{i}\pi_{j}+z_{i}y_{i0}^{\left(\text{hp}\right)}\eta_{j1}+z_{i}y_{i0}^{\left(\text{lp}\right)}\eta_{j2}+z_{i}y_{i0}^{\left(\text{ue-short}\right)}\eta_{j3}+\sigma_{\alpha_{j}}\alpha_{j}^{*}$$

with $\alpha_{j}^{*}=\alpha_{j}/\sigma_{\alpha_{j}}$. The individual likelihood contribution is:

$$L_{i}=\int_{\alpha_{1}^{*}}^{}\int_{\alpha_{2}^{*}}^{}\{\prod_{t=1}^{T}P_{it}(\alpha_{1}^{*},\alpha_{2}^{*})\}g(\alpha_{1}^{*})g(\alpha_{2}^{*})\text{d}\alpha_{1}^{*}\text{d}\alpha_{2}^{*}$$ (7)

and $g(\alpha_{j}^{*})$ are the probability density functions which need to be integrated out. Using random numbers based on primes numbers (also called Halton draws, see [63]), two times *R* standard uniform distributed draws ${\tilde{\alpha }}_{j}^{r}\in \left\{0,\ldots ,1\right\}$ are derived and transformed by the inverse cumulative standard normal distribution $\Phi^{-1}({\tilde{\alpha }}_{j}^{r})$. In the simulation, $\alpha_{1}^{r}=\sigma_{\alpha_{1}}{\tilde{\alpha }}_{1}^{r}$ and $\alpha_{2}^{r}=\sigma_{\alpha_{1}}\rho_{\alpha }{\tilde{\alpha }}_{1}^{r}+\sigma_{\alpha_{2}}\sqrt{1-\rho_{\alpha }^{2}}{\tilde{\alpha }}_{2}^{r}$ (see [64]). For each draw, the likelihood is derived for each observation, multiplied over all individuals and time-points and finally averaged over all draws:

$$MSL=\prod_{i=1}^{N}\frac{1}{R}\sum_{r=1}^{R}\{\prod_{t=1}^{T}P_{it}(\alpha_{1}^{r},\alpha_{2}^{r})\}$$ (8)

In this application, we use 75 Halton draws. This concludes the full description of our baseline econometric model. All estimations are undertaken in the statistical data analysis program Stata 13.1 and using the command **bireprob** ([65]).

We report coefficients from probit models, and seeing as these do not lend themselves easily to interpretation, we will also report the partial effects of low-pay employment. Average partial effects (APE) may be interpreted as the difference in percentage points of becoming unemployed, respectively higher-paid employed, between someone who was low-paid employed in the previous period compared to someone who was unemployed. Following Steward’s [20] suggestion we first derive the partial effects for each individual and then calculate the mean over the sample (see Appendix A.2). We do this separately for various local unemployment rates. Indication for a steppingstone effect of low-pay is found if the unemployment risk is reduced and the chances of becoming higher-paid employed are increased.

So far, it is assumed that the effect of the local unemployment rate on the labour market prospects is homogeneous and independent of the labour market position. However, there exists empirical evidence that, in labour markets with a low unemployment rate, the re-employment prospects of the short-term unemployed are substantially better than those for the long-term unemployed, but that the prospects of the former deteriorate more sharply when local unemployment increases ([40]). It may be that this also applies to the steppingstone effect of low pay. To explore this empirically, we first interacted the lagged labour market position with the lagged local unemployment rate (Model 2). Next, we split neighbourhoods into those with a high or low unemployment rate whereby a neighbourhood is labelled “high unemployment” if it belongs to the 40^th^ percentile of the distribution with the highest unemployment rates, and “low unemployment” otherwise. Alternatively, for the range between the 60^th^ and 80^th^ percentile of the distribution with the lowest unemployment rates, we have estimated for each percentage point an uncorrelated random-effects probit model and took as threshold the model with the highest cumulated log-likelihood (see S1 Fig). Afterwards, this local unemployment rate indicator for *t-1* is interacted with the lagged labour market position (Model 3).

### Further explorations

Without further restrictions, any neighbourhood effect that we identify will be driven by neighbourhoods transitioning into or out of low local unemployment over time, by people moving to a different neighbourhood, and, in the case of LSOA, by neighbourhood boundary changes. As LSOA boundaries are drawn so they are socially homogenous localities, nested within local authorities and not exceeding a population threshold of 3,000 people (for further information on boundary changes, see http://www.ons.gov.uk/ons/guide-method/geography/beginner-s-guide/census/output-area--oas-/index.html), boundary changes mark significant population change, which has occurred in some localities due to new housing development and migration. While such major structural changes in the local environment may well impact people’s employment and earnings prospects, they are not the norm and they may bias our neighbourhood effect. To address this issue, we restrict the sample to respondents whose LSOA boundaries were not redrawn between the 2001 and 2011 Censuses (Model 4); for this sample the bulk of the neighbours and the space covered in *t* and *t-1* is the same and a change in the local unemployment rate means from *t-1* to *t* means just that: that a greater (or smaller) share of neighbours are in employment. To more directly assess the extent to which unobserved neighbourhood characteristics may be driving the results, we then restrict the sample to respondents who live in the same neighbourhood throughout the observation period (thus netting-out the neighbourhood fixed effect) (Model 5). To minimize the effect of unobserved local characteristics as well as major local change, we then drop both movers and those who live in an LSOA whose boundary has changed (Model 6).

Last but not least, we exclude from the sample individuals who have post-secondary education seeing as they have been shown to face lower risks of unemployment and low-pay. Whilst we control for education in our baseline model, differences may be more structural; we expect stronger persistence in unemployment and low-pay employment (Model 7).

## Results

### The baseline model

Results of our main analysis are presented in Table 3. We report relevant information on the random effects parameters (bottom panel), the coefficients for the respondent’s lagged labour market position and the lagged unemployment rate in the neighbourhood as they are required to derive the steppingstone effect of low pay (top panel; see S5 Table for complete estimation results of Models 1–3. To alleviate the concern that the neighbourhood boundaries may be drawn too tightly, we also replaced the local unemployment rate at LSOA-level by unemployment rates at the much more aggregated scales of regions (GOR) and local authority districts (LAD), respectively. Model comparisons on the basis of information criteria and the share of correct predictions support our hypothesis that the effect is best measured at the LSOA-level, see S6 Table). The first two columns present the results for Model 1 (which includes the local unemployment rate in continuous form), followed by the results for Model 2 (which relaxes the assumption of effect heterogeneity for different labour market positions), and the results for Model 3 (which includes a dichotomous indicator for low vs. high local unemployment). Within each of the model results, the first column refers to the probability of becoming unemployed and the second column to the conditional probability of becoming higher-paid employed. The reference category is being long-term unemployed.

**Table 3:** Correlated random effects probit regression of lagged labour market position on current labour market position.

Dependent variable:	*ue*_*t*_	*hp*_*t*_	*ue*_*t*_	*hp*_*t*_	*ue*_*t*_	*hp*_*t*_
Model	Model 1		Model 2		Model 3	
higher-pay_*t*−1_	-1.635^‡^ (0.271)	1.189^‡^ (0.407)	-1.529^‡^ (0.359)	1.077 (0.810)	-1.524^‡^ (0.339)	0.842 (0.613)
low-pay_*t*−1_	-1.592^‡^ (0.243)	0.362 (0.416)	-1.479^‡^ (0.358)	0.134 (0.818)	-1.486^‡^ (0.339)	-0.004 (0.623)
short-term unemployed_*t*−1_	-0.808^‡^ (0.211)	0.198 (0.436)	-0.796^‡^ (0.372)	0.067 (0.865)	-1.100^‡^ (0.346)	-0.027 (0.664)
long-term unemployed_*t*−1_	*reference category*					
ue-rate_*t*−1_	6.151^‡^ (1.869)	-7.122^‡^ (2.017)				
ue-rate_*t*−1_ × higher-pay_*t*−1_			5.604^†^ (2.482)	-8.265^‡^ (2.339)		
ue-rate_*t*−1_ × low-pay_*t*−1_			5.716* (3.009)	-5.185* (2.682)		
ue-rate_*t*−1_ × short-term unemployed_*t*−1_			8.217* (4.882)	-7.711 (7.527)		
ue-rate_*t*−1_ × long-term unemployed_*t*−1_			8.214 (5.044)	-10.886 (17.357)		
ue-high_*t*−1_ × higher-pay_*t*−1_					0.171 (0.104)	-0.285^‡^ (0.096)
ue-high_*t*−1_ × low-pay_*t*−1_					0.285* (0.158)	-0.274^†^ (0.121)
ue-high_*t*−1_ × short-term unemployed_*t*−1_					0.907^‡^ (0.273)	-0.640 (0.397)
ue-high_*t*−1_ × long-term unemployed_*t*−1_					0.444 (0.273)	-0.905 (0.722)
Controls	included		included		included	
$\sigma_{\alpha_{1}}^{2}$	0.351^†^ (0.163)		0.349^†^ (0.162)		0.328^†^ (0.161)	
$\sigma_{\alpha_{2}}^{2}$	1.324^‡^ (0.227)		1.302^‡^ (0.224)		1.296^‡^ (0.225)	
*ρ*_*α*_	-0.283* (0.159)		-0.279* (0.159)		-0.240 (0.157)	
AIC	*5785*.*6039*		*5796*.*0016*		*5788*.*9857*	
BIC	*6372*.*8651*		*6425*.*7154*		*6418*.*6995*	
per cent of correct predictions	*87*.*60*		*87*.*70*		*87*.*75*	
LR-test^a^	*χ*^*2*^(2) = 22.44 [p-*val* < 0.001]		*χ*^*2*^(2) = 24.04 [p-*val* < 0.001]		*χ*^*2*^(2) = 31.06 [p-*val* < 0.001]	
log likelihood	*-2809*.*802*		*-2809*.*001*		*-2805*.*493*	
*N*	*8*,*738*		*8*,*738*		*8*,*738*	

*Source*: Understanding Society (2015), Waves 1–5, 2009–2014, linked with DfT Accessibility Statistics 2013. Standard errors in parenthesis, levels of significance:

* *p* < .10;

^†^ *p* < .05;

^‡^ *p* < 0.1. For complete estimation results see S5 Table.

^*a*^ Reference model excludes local unemployment rate indicator.

*Model 1* includes the local unemployment rate in continuous form, *Model 2* relaxes the assumption of effect heterogeneity for different labour market positions and *Model 3* includes a dichotomous indicator for low vs. high local unemployment.

The results for Model 1 suggest that higher-pay and low-pay employment reduce the risk of becoming unemployed (column 1). The short-term unemployed face a significantly lower risk of becoming unemployed in the subsequent period than the long-term unemployed. They do, however, also have a significantly greater risk to remain unemployed than the low-paid employed to become unemployed $\left({\text{H}}_{0}:{\hat{\gamma }}_{12}={\hat{\gamma }}_{13};\chi^{2}(1)=15.03[\text{p-}val<0.01]\right)$. Neighbourhood unemployment alters the employment prospects considerably: the risk staying or becoming unemployed rises as the local unemployment rate increases.

With respect to the conditional probability of entering higher pay (column 2), the results suggest that whilst those who are already on higher pay have a significantly higher chance to remain in this state than the long-term unemployed to enter it, the chances for those on low pay or in short-term unemployment are not markedly improved compared to those in long-term unemployment. As before, there is empirical evidence that the neighbourhood unemployment rate impacts prospects: the probability to become higher-paid employed decreases as local unemployment goes up, and this effect is significantly different from zero.

Table 3, columns 3 and 4, reports the results for Model 2, where we relax the assumption that there is a homogeneous effect of local unemployment on employment and earnings prospects for all labour market positions (i.e., by interacting the lagged local unemployment rate with the lagged labour market position). Whilst we find some indication that the employment and earnings prospects of the higher-paid employed and low-paid employed are less influenced by the local unemployment level than the short-term, resp. long-term unemployed, none of the coefficients are different from each other (in terms of conventional levels of statistical significance).

Last but not least, the results for Model 3 (Table 3, column 5 and 6) suggest that the short-term unemployed, the low-paid and higher-paid employed who live in neighbourhoods with low unemployment have the same risk of becoming unemployed as the long-term unemployed to remain unemployed $\left({\text{H}}_{0}:{\hat{\gamma }}_{12}={\hat{\gamma }}_{13};\chi^{2}(1)=1.65[\text{p-}val=0.20];{\text{H}}_{0}:{\hat{\gamma }}_{11}={\hat{\gamma }}_{13};\chi^{2}(1)=2.16[\text{p-}val=0.14]\right)$. By contrast, the short-term unemployed who live in neighbourhoods with high unemployment have a significantly higher risk of becoming unemployed compared to their counterparts living in neighbourhoods with low unemployment (ue-high_*t*−1_ × short-term unemployed_*t*−1_ = 0.907, p-*val* < 0.01). This finding also holds for those in other labour market positions, albeit the gaps in unemployment risks are less pronounced. We also find gaps in the prospects of becoming high-paid employed for those in the same labour market position living in different types of neighbourhoods: especially the short-term and the long-term unemployed are faced with a much lower chance to become higher-paid employed in high unemployment neighbourhoods compared with their respective counterparts in neighbourhoods with low unemployment.

The random-effects parameters for Models 1–3 (bottom panel) suggest that individuals also differ in their unobserved characteristics: For instance, whilst 26.0 per cent of the variance in the error term in Model 1 is explained by the random-effects error term in the unemployment equation, 57.0 per cent of the composite variance is explained by the random-effects error term in the higher-pay equation. Moreover, the random-effects are negatively correlated (*ρ*_*α*_ = −0.283), which indicates that unobserved characteristics that positively affect higher-pay employment affect the probability of unemployment negatively.

Finally, some tests were undertaken to test each of the models and to determine which of them performs the best in statistical terms. In particular, Likelihood Ratio tests were undertaken to compare the respective models with a specification that does not account for local unemployment, indicating that inclusion of local unemployment significantly improves each model’s performance. To compare model performance across specifications, we calculated information criteria (AIC and BIC) and the share of correct predictions. Whilst Model 1 yields the best fit with respect to the information criteria, Model 3 produces the highest share of correct predictions.

**Fig 3:**
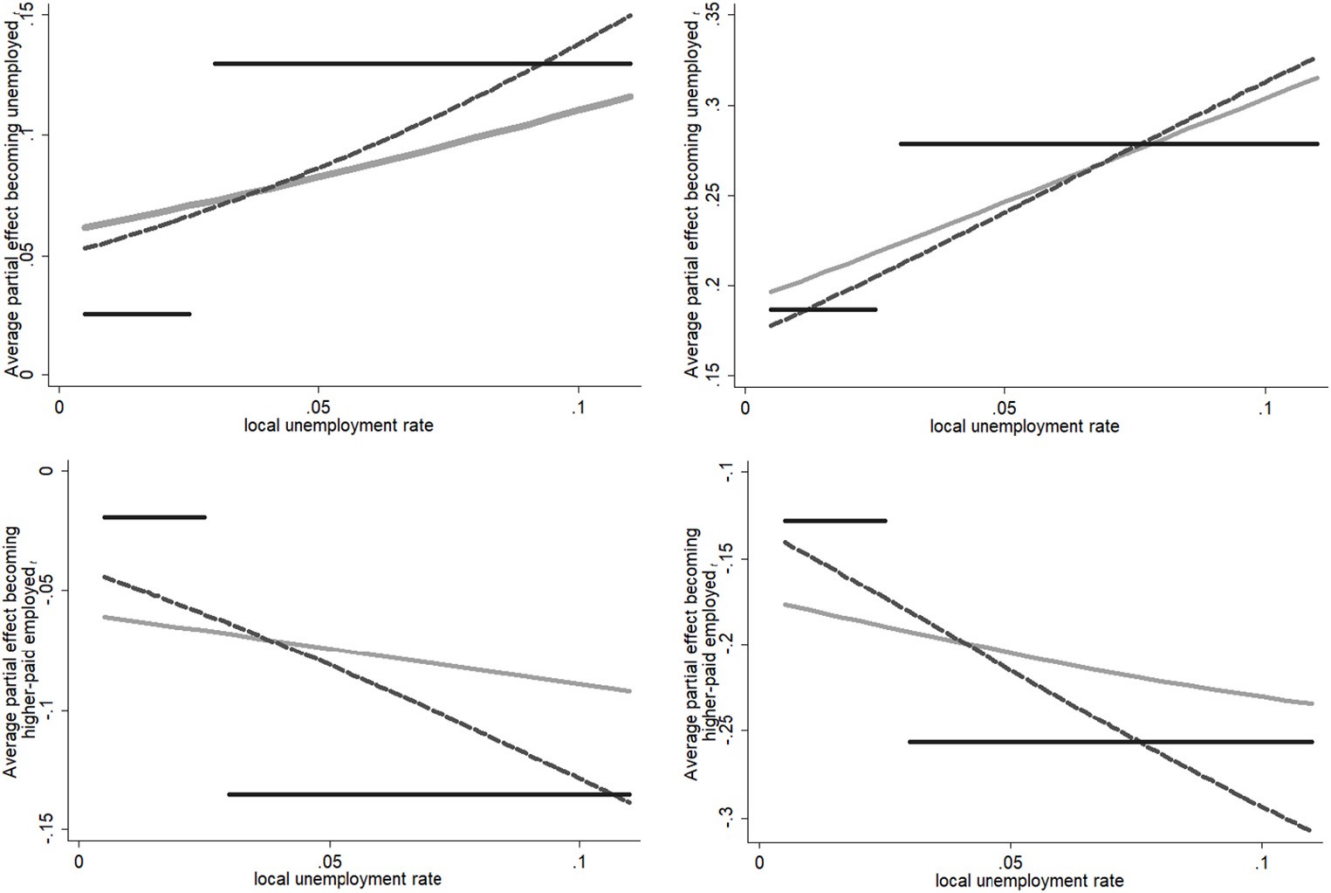
Average partial effect of becoming unemployed (upper panel), resp. higher-paid (lower panel) employed, at *t*. *Source*: Understanding Society (2015), Waves 1–5, 2009–2014; linked with DfT Accessibility Statistics 2013. The panel on the left shows the APE between someone short-term unemployed and someone low-paid employed at *t-1* of becoming unemployed (top), resp. higher-paid employed (bottom); the panel on the right refers to the long-term unemployed. *Model 1* includes the local unemployment rate in continuous form, *Model 2* relaxes the assumption of effect heterogeneity for different labour market positions and *Model 3* includes a dichotomous indicator for low vs. high local unemployment. Model (1): grey line; Model (2) dashed line; Model (3): black line.

To facilitate interpretation of the results, we also calculated the average partial effects (APE) for Models 1–3 and present the results graphically in Fig 3. Holding observable characteristics of the individual constant, the panel on the left shows the APE of becoming unemployed (top), respectively, higher-paid employed (bottom) at *t* for someone who was low paid instead of being short-term unemployed at *t-1*; the panel on the right presents the complementary figures for the long-term unemployed. APE at select levels of local unemployment are presented in S7 Table (Models 1 and 2), and for low and high unemployment neighbourhoods in Table 4 (Model 3).

**Table 4:** Average partial effects of short-term unemployed (APE).

	Model 3		Model 4		Model 5		Model 6		Model 7	
*Ue duration*_*t-1*_	Short	long	Short	long	short	Long	short	Long	Short	Long
	*Low local unemployment rate*									
Unemployed_*t*_	0.026 (0.029)	0.187* (0.112)	0.034 (0.036)	0.198 (0.125)	0.068 (0.043)	0.275^†^ (0.129)	0.033 (0.029)	0.154 (0.099)	0.004 (0.027)	0.140 (0.112)
Higher-paid_*t*_	-0.019 (0.050)	-0.128 (0.120)	-0.029 (0.056)	-0.129 (0.130)	-0.003 (0.058)	-0.111 (0.132)	0.025 (0.051)	-0.013 (0.109)	-0.001 (0.066)	-0.046 (0.140)
	*High local unemployment rate*									
Unemployed_*t*_	0.130* (0.073)	0.278^†^ (0.126)	0.133 (0.081)	0.267^†^ (0.134)	0.205^†^ (0.084)	0.356‡ (0.127)	0.111* (0.061)	0.191* (0.100)	0.099 (0.074)	0.238* (0.143)
Higher-paid_*t*_	-0.135* (0.072)	-0.256^†^ (0.110)	-0.125* (0.076)	-0.246^†^ (0.115)	-0.126 (0.080)	-0.239^†^ (0.113)	-0.064 (0.063)	-0.125 (0.100)	-0.130* (0.073)	-0.178 (0.118)
*N*	2,477	2,477	2,349	2,349	1,935	1,935	1,859	1,859	1,310	1,310

*Source*: Understanding Society (2015), Waves 1–5, 2009–2014; linked with DfT Accessibility Statistics 2013. Standard errors in parenthesis, levels of significance:

* *p* < .10;

^†^ *p* < .05;

^‡^ *p* < .01.

*Model 4*: Individuals in LSOAs without boundary change only.

*Model 5*: Non-movers only.

*Model 6*: Non-movers in LSOAs without boundary change only.

*Model 7*: Men without post-secondary education only.

While the short-term unemployed has an on average six percentage points higher risk of staying unemployed compared to someone who was on low pay when the local unemployment rate is close to zero, the implied steppingstone effect of low-pay doubles when the local unemployment rate is 10per cent. Moreover, the chances of becoming higher-paid employed are between six and nine percentage points lower for the short-term unemployed. This group’s APE of staying unemployed increases from about 20 percentage points at low levels of neighbourhood unemployment to about 30 percentage points at high levels of neighbourhood unemployment. The chances of becoming higher-paid employed, too, are, on average, between 17.7 and 23.4 percentage points lower for the long-term unemployed compared to the low-paid employed. These findings indicate that taking up low pay is instrumental in reducing the risk of future unemployment and in increasing the chances to climb up the salary ladder for the long-term and short-term unemployed, in particular when living in neighbourhoods with high unemployment.

When we account for a heterogeneous effect of local unemployment (Model 2), the impact of the local labour market condition becomes more pronounced as the slopes of the APE become steeper. We calculated the unemployment-related slope of the average partial effect, which gives the change of the APE in percentage points when local unemployment increases by 1 percentage point (S7 Table, last row). In Model 1, the probability of a short-term unemployed to stay unemployed (compared to someone low-paid employed), on average, increases by 0.52 pp. when local unemployment level grows by 1 pp.–and the slope increases to 0.92 pp. in Model 2. Moreover, for the short-term unemployed, the slope of the APE to ascend the earnings ladder changes from -0.29 if local unemployment increases by 1 pp. to -0.91 pp. in Model 2. In the case of long-term unemployed, a noticeable steeper slope (in absolute values) is detected for the probability of becoming higher-paid employed: while in Model 1 the chances of someone long-term unemployed drops, on average, by 0.55 pp. if the local unemployment rate increases by 1 pp., it is 1.62 pp. in Model 2.

The heterogeneous effect of local unemployment also manifests itself in Model 3: while the risk of becoming unemployed is, on average, 2.6 percentage points higher for the short-term unemployed than for the low-paid employed in neighbourhoods with low unemployment, the risk amounts to 13.0 percentage points in neighbourhoods with high unemployment. Furthermore, while the short-term unemployed have a slightly lower chance of becoming higher-paid employed (-1.9 percentage points) in low unemployment neighbourhoods, the APE amounts to -13.5 pp in high unemployment neighbourhoods. Changes in the APEs implied by moving from the lowest to highest unemployment neighbourhoods for the long-term unemployed are comparable, suggesting that this group’s odds of leaving unemployment and becoming higher-paid employed are considerably lower than those of the low-paid employed as the level of local unemployment level.

Overall, then, we find heterogeneity in labour market state dependence conditional on the unemployment level in the neighbourhood. The results suggest that there is a steppingstone effect of low pay, which is particularly marked in neighbourhoods with high unemployment. In these places, the risk of future unemployment is lowered, and the prospects of becoming higher-paid employed are substantially increased when individuals enter low-pay employment compared to when they remain unemployed.

### Further explorations

With respect to testing the steppingstone effect across neighbourhoods (see Table 4), we hypothesized that the local labour market effects that we observe from Model 3 may be driven by unobserved neighbourhood characteristics. However, dropping individuals in neighbourhoods with LSOA boundary changes (Model 4) does not produce effects that are statistically different from the baseline model. We furthermore argued that other unobserved neighbourhood characteristics may be driving the results and therefore restricted the sample to non-movers only (Model 5). The baseline results are confirmed in this sample, too. The results are also confirmed when we combine the restrictions from Models 4 and 5 (Model 6), albeit the APE of becoming higher-paid employed are considerably smaller than those implied by Model 3.

Our final test, see Model 7, shows little in support of the hypothesis that there are structural differences in the effects for those with and without post-secondary education which cannot be captured by including a respective indicator variable in the regression. The key results from the baseline are replicated in a sample of only those with post-secondary education.

## Conclusion

Against the backlight of rising unemployment and a persistent high share of low-pay employment in many countries ([15]), the aim of this research was to provide empirical evidence on whether the employment and earnings prospects of low-pay workers compared to the unemployed are improved, and how these effects–referred to as steppingstone effects—are correlated with local labour market conditions. Whilst a plethora of studies have suggested that there is state dependence in labour market processes, few have considered that opportunities for economic advancement are not distributed evenly across space. Are those who live in neighbourhoods that are characterized by high unemployment well advised to “get on their bikes” and take a job at low pay? Taking a job could mitigate the hypothesized deterioration in human capital experienced during unemployment–in particular for the long-term unemployed–and low-pay workers may gain new skills which could help reduce their future unemployment risk and improve their earnings prospects. Or should they hang on and invest their time into looking for a better-paid job?

To investigate these issues empirically, we use information from the first five waves of interviews from Understanding Society, the UK Household Longitudinal Study (UKHLS). The study is representative for all regions of the United Kingdom and for rural and urban areas within them. We matched the panel data with official statistics for neighbourhoods in England, providing local unemployment rates which we use as an indicator for how much competition those in the low-wage sector may experience: Compared to those working at higher pay, low-paid workers tend to work much more locally, and the unemployment rate that we employ is calculated much more locally than previous research was able to observe, and, it appears, at a level where it really does matter.

The empirical results suggest that there are differences in the returns to taking up low pay depending on where one lives. In areas with a high local unemployment rate, we find strong indication for a steppingstone effect of low pay. Not only is the risk of future unemployment reduced for those who work on low pay but also is the probability of becoming higher-paid employed substantially improved. A possible explanation is that the value of signalling one′s willingness to work is positively correlated with the number of unemployed people in the neighbourhood, and with the scarcity of higher-paid jobs.

We find that the steppingstone effects vary according to how long unemployment has lasted: Whilst there is a weak steppingstone effect of low pay for the short-term unemployed living in neighbourhoods with low unemployment, the effects are sizeable and statistically robust across several specifications in neighbourhoods with high unemployment. The steppingstone effect of low pay is larger, however, for the long-term unemployed and its size increases considerably as local unemployment rises.

There are a number of challenges in identifying these local interaction effects, and we address these inasmuch as is feasible. One of the paramount challenges is that there may be unobserved neighbourhood characteristics that are correlated with employment opportunities and people’s location choice. Economically attractive areas, for example, tend to receive a lot of investment, new properties are being built, and people move in; the results of our robustness tests suggest that these factors are not driving our results. Whilst our findings hold for a number of alternative model specifications, including when unobserved neighbourhood characteristics are controlled for, they are not fully in support of Steward’s [20] conclusion “that low-wage jobs typically do not lead on to better things” [p. 529]. Whilst the author finds no significant differences between the low-paid employed and unemployed in the risk of becoming unemployed, our study shows that especially in high unemployment neighbourhoods low-pay employment improves the labour market prospects compared to short-term and long-term unemployment.

In the specific population and period of time that we examined here, there clearly were some groups for whom low pay work has paid off. Albeit, qualitative research with residents in deprived neighbourhoods documents that low wages do not typically pay enough to maintain the family, and the costs associated with being in employment (e.g., commuting costs) put enormous pressure on workers who have to make ends meet (see, e.g., [66]). In this context, the current shifts in employment contracts towards reduced job stability (e.g., fixed-term contracts) and uncertain payment structures (e.g., zero-hour contracts) mean that the incentives to take up low-pay employment have been further reduced for exactly that part of the population for whom–according to our empirical study—the greatest benefits were had from engaging in the low pay sector. Potential solutions would include policies that increase job security, limit the use of zero-hours contracts and offer support for skills improvement and career advancement.

## Supporting information

S1 Fig Cumulated log-likelihood and cut-off point. *Source*: Understanding Society (2015), Waves 1–5, 2009–2014 linked with DfT Accessibility Statistics 2013. N = 8,738.
(TIF)

S1 Table Correlation matrix of the unemployment rate at LSOA level. (PDF)

S2 Table Local unemployment rate and labour market position. (PDF)

S3 Table Listing of control variables with description. (PDF)

S4 Table Descriptive statistics. (PDF)

S5 Table Correlated random effects probit regression of lagged labour market position on current labour market position (complete). (PDF)

S6 Table Estimation results for different geographical unemployment marker. (PDF)

S7 Table Average partial effects at different levels of local unemployment. (PDF)

S1 File Average partial effects. (PDF)

## References

[1] Layard R, Nickell S, Jackman R. Unemployment: Macroeconomic Performance and the Labour Market, Oxford University Press, Oxford; 1991.

[2] Mayhew K. The changing shape of the UK job market and its implications for the bottom half of earners. London: Resolution Foundation; 2012.

[3] Devins D, Bickerstaffe T, Mitchell B, Halliday S. Improving progression in low-paid, low-skilled retail, catering and care jobs. 2014.

[4] Anger S. Unpaid Overtime in Germany: Differences Between East and West. Journal of Applied Social Science Studies (Schmollers Jahrbuch). 2005;125: 17–21.

[5] Galster GC. The mechanism (s) of neighbourhood effects: Theory, evidence, and policy implications In: Neighbourhood effects research: New perspectives 2012 (pp. 23–56). Springer, Dordrecht.

[6] Arnott R, Rowse J. Peer group effects and educational attainment. Journal of Public Economics. 1987;32: 287–305.

[7] Wilson W. The truly disadvantaged: The inner city, the underclass, and public policy, Chicago: The University of Chicago 1987.

[8] Selod H, Zenou Y. City structure, job search and labour discrimination: Theory and policy implications. The Economic Journal. 2006;116: 1057–1087.

[9] Bayer P, Ross SL, Topa G. Place of work and place of residence: Informal hiring networks and labor market outcomes. Journal of Political Economy. 2008;116: 1150–1196.

[10] Zenou Y, and Boccard N. Racial discrimination and redlining in cities. Journal of Urban Economics. 2000;48: 260–285.

[11] Patacchini E, Zenou Y. Spatial dependence in local unemployment rates. Journal of Economic Geography. 2007;7: 169–191.

[12] Hoynes HW. Local labor markets and welfare spells: Do demand conditions matter? Review of Economics and Statistics. 2000;82: 351–368.

[13] Van der Klaauw B, Van Ours JC. From welfare to work: does the neighborhood matter? Journal of Public Economics. 2003;87: 957–985.

[14] Åberg Y, Hedström P, Kolm AS. Social interactions and unemployment. Nationalekonomiska institutionen; 2003.

[15] OECD. OECD Employment Outlook 2013. OECD Publishing; 2013.

[16] Veen A, Barratt T, Goods C. Platform-Capital’s ‘App-etite’for Control: A Labour Process Analysis of Food-Delivery Work in Australia. Work, Employment and Society. 2019;5.

[17] Peticca-Harris A, deGama N, Ravishankar MN. Postcapitalist precarious work and those in the ‘drivers’ seat: Exploring the motivations and lived experiences of Uber drivers in Canada. Organization: 2018.

[18] Taylor M, Marsh G, Nicol D, Broadbent P. Good work: The Taylor review of modern working practices: 2017. Retrieved from (9 August 2019): https://www.gov.uk/government/publications/good-work-the-taylor-review-of-modern-working-practices

[19] Webster J. Microworkers of the gig economy: separate and precarious. New Labor Forum. 2016;25: 56–64.

[20] Stewart M. The interrelated dynamics of unemployment and low-wage employment. Journal of Applied Econometrics. 2007;22: 511–531.

[21] Plum A. The British low-wage sector and the employment prospects of the unemployed. Applied Economics. 2019;51: 1411–1432.

[22] Van Ham M, Manley D, Bailey N, Simpson L, Maclennan D. Understanding neighbourhood dynamics: New insights for neighbourhood effects research. Springer Netherlands 2013: 1–21.

[23] Manski C. Identification of endogenous social effects: The reflection problem. The Review of Economic Studies. 1993;60: 531–542.

[24] Galster GC. Quantifying the effect of neighbourhood on individuals: challenges, alternative approaches, and promising directions. Journal of Applied Social Science Studies (Schmollers Jahrbuch). 2008;128: 7–48.

[25] Heckman JJ. Heterogeneity and state dependence In: Studies in labor markets (pp. 91–140). University of Chicago Press.

[26] Heckman JJ. The incidental parameters problem and the problem of initial conditions in estimating a discrete time-discrete data stochastic process and some Monte Carlo evidence. University of Chicago Center for Mathematical studies in Business and Economics; 1987.

[27] Knies G, Burgess S, Propper C. Keeping up with the Schmidt’s: An empirical test of relative deprivation theory in the neighbourhood context. Schmollers Jahrbuch: Journal of Applied Social Science Studies. 2008;128: 75–108.

[28] Vishwanath T. Job search, stigma effect, and escape rate from unemployment. Journal of Labor Economics. 1989;7: 487–502.

[29] Blanchard O, Diamond P. Ranking, unemployment duration, and wages. The Review of Economic Studies. 1994;61: 417–434.

[30] Acemoglu D. Public policy in a model of long-term unemployment. Economica. 1995;1: 161–78.

[31] Pissarides C. Equilibrium unemployment theory, MIT Press, Cambridge: 1990.

[32] Heckman JJ, Borjas G. Does unemployment cause future unemployment? Definitions, questions and answers from a continuous time model of heterogeneity and state dependence. Economica. 1980;47: 247–283.

[33] Arulampalam W, Booth A, Taylor M. Unemployment persistence. Oxford Economic Papers. 2000;52: 24–50.

[34] Mühleisen M, Zimmermann K. A panel analysis of job changes and unemployment. European Economic Review. 1994; 38: 793–801.

[35] Doiron D, Gørgens T. State dependence in youth labor market experiences, and the evaluation of policy interventions. Journal of Econometrics. 2008;145: 81–97.

[36] Ayllón S. Unemployment persistence: not only stigma but discouragement too. Applied Economics Letters. 2013;20: 67–71.

[37] Raaum O, Røed K. Do business cycle conditions at the time of labor market entry affect future employment prospects? Review of Economics and Statistics. 2006;88: 193–210.

[38] Plum A, Ayllón S. Heterogeneity in unemployment state dependence."Economics Letters. 2015:136: 85–87.

[39] Oberholzer-Gee F. Nonemployment stigma as rational herding: A field experiment. Journal of Economic Behavior & Organization. 2008;65: 30–40.

[40] Kroft K, Lange F, Notowidigdo M. Duration dependence and labor market conditions: Evidence from a field experiment. The Quarterly Journal of Economics. 2013;128: 1123–1167.

[41] Eriksson S, Rooth D. Do employers use unemployment as a sorting criterion when hiring? Evidence from a field experiment. American Economic Review. 2014;104: 1014–1039.

[42] Van den Berg GJ, Van Ours JC. Unemployment dynamics and duration dependence. Journal of Labor Economics. 1996;14: 100–25.

[43] Imbens GW, Lynch LM. Re-employment probabilities over the business cycle. Portuguese Economic Journal. 2006;5: 111–34.

[44] McCormick B. A theory of signaling during job search, employment efficiency, and “stigmatized” jobs. The Review of Economic Studies. 1990;57: 299–313.

[45] Hurrell A. Starting out or getting stuck: An analysis of who get trapped in low-paid work–and who escapes. London: Resolution Foundation; 2013.

[46] Uhlendorff A. From no pay to low pay and back again? A multi-state model of low pay dynamics. IZA Discussion Papers, Institute for the Study of Labor (IZA): 2006.

[47] Knabe A, Plum A. Low-wage jobs–springboard to high-paid ones? Labour: Review of Labour Economics and Industrial Relations. 2013;27: 310–330.

[48] Mosthaf A. Do scarring effects of low-wage employment and non-employment differ between levels of qualification? Scottish Journal of Political Economy. 2014;61: 154–177.

[49] Mosthaf A, Schank T, Schnabel C. Low-wage employment versus unemployment: Which one provides better prospects for women? IZA Journal of European Labor Studies. 2014;3: 1–21

[50] Buddelmeyer H, Lee WS, Wooden M. Low-paid employment and unemployment dynamics in Australia. Economic Record. 2010;86: 28–48.

[51] Cappellari L. Earnings mobility among Italian low-paid workers. Journal of Population Economics. 2007;20: 465–482.

[52] Clark K, Kanellopoulos N. Low pay persistence in Europe. Labour Economics. 2013;23: 122–134.

[53] Galster GC, Killen SP. The geography of metropolitan opportunity: a reconnaissance and conceptual framework. Housing Policy Debate. 1995;6: 7–43.

[54] Akerlof GA. A theory of social custom, of which unemployment may be one consequence. The Quarterly Journal of Economics. 1980;94: 749–75.

[55] Streufert P. The effect of underclass social isolation on schooling choice. Journal of Public Economic Theory. 2000;2: 461–482.

[56] University of Essex: 2015: Institute for Social and Economic Research and National Centre for Social Research, Understanding Society: Waves 1–5, 2009–2014 [computer file].

[57] Knies G. Understanding Society—UK Household Longitudinal Study: Wave 1–5, 2009–2014. User Manual, Colchester: University of Essex; 2015.

[58] OECD. OECD Employment Outlook 1997—Low-wage jobs: stepping stones to a better future or traps? OECD Publishing; 1997.

[59] Sutherland A, Brunton-Smith I, Jackson J. Collective Efficacy, Deprivation and Violence in London. The British Journal of Criminology. 2013;53: 1050–1074.

[60] Murphy E. Measuring Employment and Unemployment. In: Northern Ireland Assembly (ed) Research and Information Service Briefing Note. 2013

[61] Knies G, Menon S. Understanding Society: Waves 1–3, 2009–2012: Special license access, Geographical Accessibility, User Guide. Technical report, Understanding Society at the Institute for Social and Economic Research; 2014. Published online: https://goo.gl/kAERBO

[62] Wooldridge J. Simple solutions to the initial conditions problem in dynamic, nonlinear panel data models with unobserved heterogeneity. Journal of Applied Econometrics. 2005;20: 39–54.

[63] Train KE. Discrete Choice Methods with Simulation. Cambridge University Press, Cambridge: 2009.

[64] Alessie R, Hochguertel S, van Soest A. Ownership of stocks and mutual funds: A panel data analysis. Tilburg University; 2001.

[65] Plum A. bireprob: An estimator for bivariate Random-Effects Probit Models. Stata Journal: 2016;16: 96–111.

[66] Open Society Foundations. Europe’s White working class–Manchester. Open Society Foundations: New York Online: http://goo.gl/7jMo7s: 2014.

