# Supplementary figures and images for: Local unemployment changes the springboard effect of low pay: Evidence from England
Source: PLoS One. 2019 Nov 13;14(11):e0224290. doi: 10.1371/journal.pone.0224290 (PMC6853294; doi:10.1371/journal.pone.0224290)

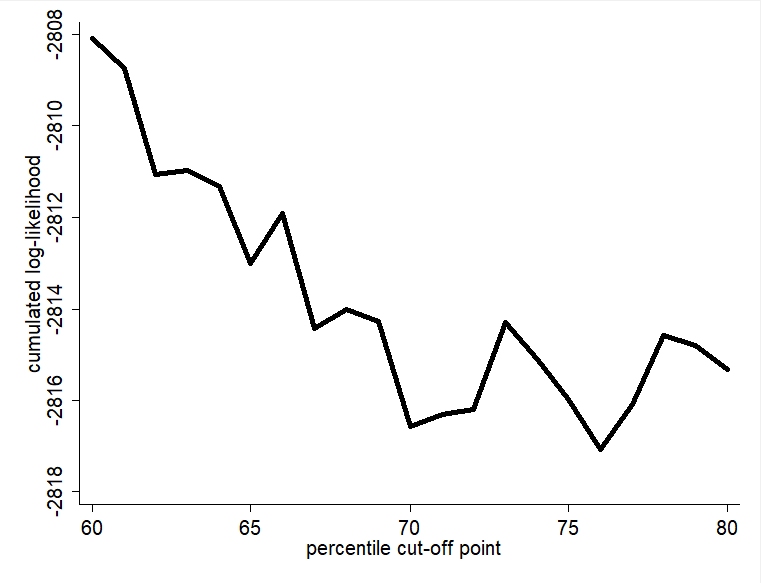

Supplement: S1 Fig — Source: Understanding Society (2015), Waves 1–5, 2009–2014 linked with DfT Accessibility Statistics 2013. N = 8,738. (TIF) [file pone.0224290.s001.tif]
