# Supplementary material for: Local unemployment changes the springboard effect of low pay: Evidence from England
Source: PLoS One. 2019 Nov 13;14(11):e0224290. doi: 10.1371/journal.pone.0224290 (PMC6853294; doi:10.1371/journal.pone.0224290)
Supplement: S1 Table — (PDF) [file pone.0224290.s002.pdf]

S1 Table: Correlation matrix of the unemployment rate at LSOA level

Year	2009	2010	2011	2012	2013
2009	1.0000				
2010	0.9334	1.0000			
2011	0.9218	0.9022	1.0000		
2012	0.9721	0.9158	0.9138	1.0000	
2013	0.9505	0.8892	0.8945	0.9815	1.0000

Source: DfT Accessibility Statistics 2013. The table shows the correlation matrix of the unemployment rate on LSOA level between different years.
