# Supplementary material for: Local unemployment changes the springboard effect of low pay: Evidence from England
Source: PLoS One. 2019 Nov 13;14(11):e0224290. doi: 10.1371/journal.pone.0224290 (PMC6853294; doi:10.1371/journal.pone.0224290)
Supplement: S2 Table — (PDF) [file pone.0224290.s003.pdf]

S2 Table. Local unemployment rate and labour market position

Unemployment quartile	1st	2nd	3rd	4th
Higher-Paid$_t$	28.6	27.2	24.9	19.4
Low-Paid$_t$	13.7	18.9	26.0	41.4
Short-term unemployed$_t$	17.7	17.1	26.9	38.3
Long-term unemployed$_t$	7.3	7.3	23.9	61.5
Total$_t$	25.1	24.9	25.1	24.9

Source: Understanding Society (2015), Waves 1-5, 2009-2014; linked with DfT Accessibility Statistics 2013. N=8,738.

Pearson $\chi^2(9) = 621.37$ (p-value $< .001$).
