# Supplementary material for: Local unemployment changes the springboard effect of low pay: Evidence from England
Source: PLoS One. 2019 Nov 13;14(11):e0224290. doi: 10.1371/journal.pone.0224290 (PMC6853294; doi:10.1371/journal.pone.0224290)
Supplement: S3 Table — (PDF) [file pone.0224290.s004.pdf]

S3 Table. Listing of Control variables with description

Variables	*Description*
Young	Dummy: 1 if the person is 30 or below, 0 otherwise.
Old	Dummy: 1 if the person is 50 or above, 0 otherwise.
Health limits work	Categorical: between 1 (all of the time) and 5 (none of the time)
Married	Dummy: 1 if married, 0 otherwise
Child in HH	At least one child in the household
Low-unemployment region	Dummy: 1 if living in East, South-East or South-West of England and 0 otherwise
ntsarea 1	Dummy: 1 if the individual is living in (1) Inner London, (2) Outer London built-up areas, (3) West Midlands, (4) Greater Manchester, 0 otherwise
ntsarea 2	Dummy: 1 if the individual is living in (5) West Yorkshire, (7) Liverpool, (8) Tyneside, (9) South Yorkshire, (10+11) urban area over 100k population, 0 otherwise
ntsarea 3	Dummy: 1 if the individual is living in an area below 100k population, 0 otherwise
ue-rate	Continuous marker on the neighbourhood unemployment rate
Post-sec. educ.	Dummy: 1 if the individual has a degree or other higher degree, 0 otherwise
UK white	Dummy: 1 if the individual belongs to one of the following ethnic groups: white and British/English/Scottish/Welsh/Northern Irish, 0 otherwise
