# Supplementary material for: Local unemployment changes the springboard effect of low pay: Evidence from England
Source: PLoS One. 2019 Nov 13;14(11):e0224290. doi: 10.1371/journal.pone.0224290 (PMC6853294; doi:10.1371/journal.pone.0224290)
Supplement: S4 Table — (PDF) [file pone.0224290.s005.pdf]

S4 Table. Descriptive statistics

	$Total_t$	$Higher\text{-}Paid_t$	$Low\text{-}Paid_t$	$Unemployed_t$
Child in HH	0.497	0.511	0.475	0.365
Young	0.113	0.110	0.115	0.156
Old	0.170	0.162	0.193	0.213
Married	0.624	0.661	0.560	0.270
Health limits work: 1	0.009	0.008	0.012	0.04
Health limits work: 2	0.024	0.017	0.039	0.071
Health limits work: 3	0.079	0.069	0.107	0.133
Health limits work: 4	0.168	0.166	0.175	0.166
Health limits work: 5	0.720	0.740	0.667	0.59
Post-sec. educ.	0.475	0.563	0.167	0.218
UK white	0.829	0.838	0.811	0.758
NTS area: 1	0.215	0.218	0.195	0.251
NTS area: 2	0.316	0.305	0.334	0.415
NTS area: 3	0.469	0.477	0.471	0.334
Observations	*8,738*	*6,742*	*1,574*	*422*

Source: Understanding Society (2015), Waves 1-5, 2009-2014; linked with DfT Accessibility Statistics 2013.
