# Supplementary material for: Local unemployment changes the springboard effect of low pay: Evidence from England
Source: PLoS One. 2019 Nov 13;14(11):e0224290. doi: 10.1371/journal.pone.0224290 (PMC6853294; doi:10.1371/journal.pone.0224290)
Supplement: S5 Table — (PDF) [file pone.0224290.s006.pdf]

S5 Table. Correlated random effects probit regression of lagged labour market position on current labour market position (complete)

Dependent variable:	ue_t	hp_t	ue_t	hp_t	ue_t	hp_t
**Model**	**Model (1)**		**Model (2)**		**Model (3)**	
higher-pay$_{t-1}$	-1.635‡	1.189‡	-1.529‡	1.077	-1.524‡	0.842
	(0.271)	(0.407)	(0.359)	(0.810)	(0.339)	(0.613)
low-pay$_{t-1}$	-1.592‡	0.362	-1.479‡	0.134	-1.486‡	-0.004
	(0.243)	(0.416)	(0.358)	(0.818)	(0.339)	(0.623)
short-term unemployed$_{t-1}$	-0.808‡	0.198	-0.796†	0.067	-1.100‡	-0.027
	(0.211)	(0.436)	(0.372)	(0.865)	(0.346)	(0.664)
long-term unemployed$_{t-1}$			*reference category*			
ue-rate$_{t-1}$	6.151‡	-7.122‡				
	(1.869)	(2.017)				
ue-rate$_{t-1}$ × higher-pay$_{t-1}$			5.604†	-8.265‡		
			(2.482)	(2.339)		
ue-rate$_{t-1}$ × low-pay$_{t-1}$			5.716*	-5.185*		
			(3.009)	(2.682)		
ue-rate$_{t-1}$ × short-term unemployed$_{t-1}$			8.217*	-7.711		
			(4.882)	(7.527)		
ue-rate$_{t-1}$ × long-term unemployed$_{t-1}$			8.214	-10.886		
			(5.044)	(17.357)		
ue-high$_{t-1}$ × higher-pay$_{t-1}$					0.171	-0.285‡
					(0.104)	(0.096)
ue-high$_{t-1}$ × low-pay$_{t-1}$					0.285*	-0.274†
					(0.158)	(0.121)
ue-high$_{t-1}$ × short-term unemployed$_{t-1}$					0.907‡	-0.640
					(0.273)	(0.397)
ue-high$_{t-1}$ × long-term unemployed$_{t-1}$					0.444	-0.905
					(0.273)	(0.722)
Initial labour market position –(incl. interaction terms)						
higher-pay$_{t=0}$	-1.952	3.582	-1.925	3.54	-1.833	3.442
	(0.473)	(0.996)	(0.474)	(0.994)	(0.469)	(0.996)
low-pay$_{t-1}$	-1.410	1.419	-1.393	1.37	-1.348	1.271
	(0.437)	(0.979)	(0.438)	(0.978)	(0.434)	(0.979)
short-term unemployed$_{t=0}$	-0.361	1.976	-0.36	1.958	-0.348	1.917
	(0.446)	(1.165)	(0.447)	(1.162)	(0.442)	(1.152)
higher-pay$_{t=0}$ × *post-sec. educ.*	0.127	-1.534	0.098	-1.507	0.101	-1.484
	(0.346)	(0.794)	(0.351)	(0.798)	(0.348)	(0.803)
higher-pay$_{t=0}$ × UK white	0.521	-0.603	0.505	-0.579	0.444	-0.46
	(0.33)	(0.927)	(0.331)	(0.932)	(0.328)	(0.94)
low-pay$_{t=0}$ × *post-sec. educ.*	-0.116	-1.687	-0.141	-1.648	-0.137	-1.64
	(0.42)	(0.81)	(0.424)	(0.814)	(0.422)	(0.819)
low-pay$_{t=0}$ × UK white	0.109	-0.891	0.097	-0.837	0.053	-0.725
	(0.355)	(0.938)	(0.356)	(0.943)	(0.353)	(0.951)
short-term unemployed$_{t=0}$ × *post-sec. educ.*	-1.351	-1.756	-1.37	-1.744	-1.491	-1.665
	(0.663)	(1.065)	(0.664)	(1.062)	(0.681)	(1.068)
short-term unemployed$_{t=0}$ × UK white	0.389	-0.6	0.392	-0.569	0.403	-0.503
	(0.511)	(1.17)	(0.513)	(1.175)	(0.511)	(1.173)
Explanatory variables						
Child in HH	-0.208	-0.121	-0.207	-0.121	-0.207	-0.133
	(0.222)	(0.167)	(0.223)	(0.167)	(0.222)	(0.166)
Young	0.114	-0.009	0.114	-0.013	0.106	0.002
	(0.256)	(0.218)	(0.256)	(0.217)	(0.255)	(0.217)
Old	0.203	-0.426†	0.203	-0.427†	0.215	-0.442†
	(0.239)	(0.196)	(0.239)	(0.195)	(0.238)	(0.195)
Married	0.223	-0.33	0.222	-0.327	0.237	-0.32
	(0.286)	(0.237)	(0.285)	(0.237)	(0.282)	(0.237)
Health limits work: 2	0.155	-0.268	0.156	-0.291	0.169	-0.282
	(0.389)	(0.367)	(0.39)	(0.367)	(0.388)	(0.366)
Health limits work: 3	0.205	-0.333	0.208	-0.348	0.24	-0.348
	(0.375)	(0.342)	(0.376)	(0.342)	(0.374)	(0.342)
Health limits work: 4	-0.251	-0.122	-0.246	-0.141	-0.222	-0.148
	(0.37)	(0.336)	(0.371)	(0.336)	(0.368)	(0.336)
Health limits work: 5	0.002	-0.2	0.004	-0.219	0.02	-0.219
	(0.363)	(0.33)	(0.364)	(0.331)	(0.361)	(0.331)

Post-sec. educ.	-0.213	2.543‡	-0.186	2.506‡	-0.192	2.49‡
	(0.333)	(0.794)	(0.338)	(0.798)	(0.335)	(0.804)
UK white	-0.303	0.959	-0.292	0.923	-0.249	0.822
	(0.299)	(0.918)	(0.299)	(0.923)	(0.297)	(0.931)
Low-unemployment region	-0.125	0.08	-0.125	0.079	-0.132	0.088
	(0.088)	(0.094)	(0.088)	(0.094)	(0.087)	(0.093)
NTS area 2	0.532	0.483	0.525	0.483	0.52	0.456
	(0.814)	(0.852)	(0.812)	(0.852)	(0.804)	(0.844)
NTS area 3	1.055	0.975	1.046	0.97	1.006	0.976
	(0.782)	(0.774)	(0.78)	(0.775)	(0.77)	(0.766)
Year: 2011	0.104	0.037	0.103	0.037	0.09	0.053
	(0.105)	(0.084)	(0.105)	(0.084)	(0.104)	(0.083)
Year: 2012	-0.016	0.079	-0.017	0.078	-0.036	0.096
	(0.11)	(0.086)	(0.11)	(0.086)	(0.109)	(0.086)
Year: 2013	-0.095	0.056	-0.096	0.056	-0.096	0.057
	(0.117)	(0.091)	(0.117)	(0.091)	(0.116)	(0.091)
Means of explanatory variables						
average(Child in HH)	0.369	-0.076	0.366	-0.076	0.359	-0.063
	(0.248)	(0.199)	(0.249)	(0.199)	(0.247)	(0.198)
average(Young)	-0.061	0.035	-0.064	0.039	-0.049	0.024
	(0.28)	(0.257)	(0.281)	(0.256)	(0.279)	(0.256)
average(Old)	0.112	0.255	0.113	0.257	0.103	0.273
	(0.28)	(0.249)	(0.279)	(0.249)	(0.278)	(0.248)
average(Married)	-0.847‡	0.66†	-0.846‡	0.654†	-0.85‡	0.645†
	(0.311)	(0.263)	(0.311)	(0.262)	(0.308)	(0.262)
average(Health limits work: 2)	0.044	-1.072	0.062	-1.077	0.063	-1.128
	(0.745)	(1.018)	(0.747)	(1.013)	(0.735)	(1.014)
average(Health limits work: 3)	-1.165*	-0.172	-1.151*	-0.182	-1.252*	-0.166
	(0.647)	(0.85)	(0.649)	(0.847)	(0.642)	(0.848)
average(Health limits work: 4)	-0.304	0.435	-0.295	0.43	-0.372	0.459
	(0.604)	(0.825)	(0.605)	(0.822)	(0.597)	(0.823)
average(Health limits work: 5)	-0.962	0.603	-0.947	0.599	-1.023	0.62
	(0.591)	(0.808)	(0.593)	(0.805)	(0.585)	(0.805)
average(ntsarea 2)	-0.499	-0.475	-0.493	-0.473	-0.483	-0.454
	(0.814)	(0.862)	(0.813)	(0.862)	(0.805)	(0.855)
average(ntsarea 3)	-1.014	-1.087	-1.007	-1.081	-0.968	-1.08
	(0.784)	(0.785)	(0.783)	(0.785)	(0.773)	(0.776)
Constant	1.825‡	-3.123†	1.703‡	-2.906†	1.797‡	-2.751†
	(0.556)	(1.233)	(0.616)	(1.462)	(0.593)	(1.334)
$\sigma^2_{\alpha_1}$	0.351†		0.349†		0.328†	
	(0.163)		(0.162)		(0.161)	
$\sigma^2_{\alpha_2}$	1.324‡		1.302‡		1.296‡	
	(0.227)		(0.224)		(0.225)	
ρ_α	-0.283*		-0.279*		-0.240	
	(0.159)		(0.159)		(0.157)	
log likelihood	*-2809.802*		*-2809.0008*		*-2805.4928*	
N	*8,738*		*8,738*		*8,738*	

Source: Understanding Society (2015), Waves 1-5, 2009-2014 linked with DfT Accessibility Statistics 2013. Standard errors in parenthesis, levels of significance: * $p < .10$; † $p < .05$; ‡ $p < .01$.
