# Supplementary material for: Local unemployment changes the springboard effect of low pay: Evidence from England
Source: PLoS One. 2019 Nov 13;14(11):e0224290. doi: 10.1371/journal.pone.0224290 (PMC6853294; doi:10.1371/journal.pone.0224290)
Supplement: S6 Table — (PDF) [file pone.0224290.s007.pdf]

S6 Table. Estimation results for different geographical unemployment marker

Dependent variable:	ue_t	hp_t	ue_t	hp_t	ue_t	hp_t
**Unemployment marker**	**Model (1) (LSOA)**		**Government Office Regions**		**LAD**	
higher-pay$_{t-1}$	-1.635	1.189	-1.633	1.205	-1.642	1.180
	(0.271)	(0.407)	(0.271)	(0.410)	(0.274)	(0.411)
low-pay$_{t-1}$	-1.592	0.362	-1.578	0.395	-1.585	0.367
	(0.243)	(0.416)	(0.243)	(0.419)	(0.245)	(0.420)
short-term unemployed$_{t-1}$	-0.808	0.198	-0.807	0.204	-0.804	0.176
	(0.211)	(0.436)	(0.211)	(0.440)	(0.212)	(0.439)
long-term unemployed$_{t-1}$			*reference category*			
ue-rate$_{t-1}$	6.151	-7.122	-15.501	-15.582	0.532	-3.373
	(1.869)	(2.017)	(11.824)	(12.830)	(3.978)	(4.213)
Controls	included		included		included	
$\sigma^2_{\alpha_1}$	0.351		0.377		0.367	
	(0.163)		(0.168)		(0.168)	
$\sigma^2_{\alpha_2}$	1.324		1.411		1.403	
	(0.227)		(0.239)		(0.239)	
ρ_α	-0.283		-0.293		-0.295	
	(0.159)		(0.144)		(0.154)	
AIC	*5785.6039*		*5804.5962*		*5807.3935*	
BIC	*6372.8651*		*6391.8574*		*6394.6547*	
per cent of correct predictions	*87.60*		*87.50*		*87.56*	
LR-test[a]	$\chi^2(2) = 22.44$ [p-$val < 0.001$]		$\chi^2(2) = 3.45$ [p-$val = 0.179$]		$\chi^2(2) = 0.65$ [p-$val = 0.723$]	
log likelihood	*-2809.802*		*-2819.2981*		*-2820.6967*	
N	*8,738*		*8,738*		*8,738*	

Source: Understanding Society (2015), Waves 1-5, 2009-2014 linked with DfT Accessibility Statistics 2013. Standard errors in parenthesis. [a] Reference model excludes local unemployment rate indicator.
