# Supplementary material for: Local unemployment changes the springboard effect of low pay: Evidence from England
Source: PLoS One. 2019 Nov 13;14(11):e0224290. doi: 10.1371/journal.pone.0224290 (PMC6853294; doi:10.1371/journal.pone.0224290)
Supplement: S7 Table — (PDF) [file pone.0224290.s008.pdf]

S7 Table. Average partial effects at different levels of local unemployment

	Model (1)				Model (2)			
Local ue-	*Short-term* ue_t		*Long-term* ue_t		*Short-term* ue_t		*Long-term* ue_t	
rate	ue_t	hp_t	ue_t	hp_t	ue_t	hp_t	ue_t	hp_t
0.005	0.062	-0.061	0.197	-0.177	0.053	-0.044	0.178	-0.14
	(0.042)	(0.049)	(0.106)	(0.101)	(0.043)	(0.06)	(0.108)	(0.13)
0.010	0.064	-0.062	0.202	-0.18	0.056	-0.048	0.185	-0.148
	(0.043)	(0.049)	(0.108)	(0.102)	(0.043)	(0.058)	(0.109)	(0.124)
0.015	0.066	-0.064	0.207	-0.183	0.059	-0.052	0.191	-0.157
	(0.044)	(0.05)	(0.109)	(0.102)	(0.044)	(0.056)	(0.11)	(0.118)
0.020	0.068	-0.065	0.213	-0.186	0.063	-0.056	0.198	-0.165
	(0.045)	(0.051)	(0.11)	(0.102)	(0.045)	(0.054)	(0.111)	(0.113)
0.025	0.071	-0.067	0.218	-0.189	0.066	-0.06	0.205	-0.173
	(0.047)	(0.051)	(0.111)	(0.102)	(0.046)	(0.053)	(0.111)	(0.108)
0.030	0.073	-0.068	0.224	-0.193	0.07	-0.064	0.212	-0.181
	(0.048)	(0.052)	(0.113)	(0.103)	(0.047)	(0.052)	(0.112)	(0.105)
0.035	0.075	-0.07	0.229	-0.196	0.074	-0.068	0.219	-0.189
	(0.049)	(0.052)	(0.114)	(0.103)	(0.048)	(0.052)	(0.113)	(0.103)
0.040	0.078	-0.071	0.235	-0.199	0.078	-0.072	0.226	-0.198
	(0.05)	(0.053)	(0.115)	(0.103)	(0.05)	(0.053)	(0.114)	(0.103)
0.045	0.08	-0.073	0.241	-0.202	0.082	-0.077	0.233	-0.206
	(0.051)	(0.053)	(0.116)	(0.103)	(0.052)	(0.055)	(0.116)	(0.104)
0.050	0.083	-0.074	0.246	-0.205	0.086	-0.081	0.241	-0.214
	(0.052)	(0.054)	(0.117)	(0.103)	(0.054)	(0.057)	(0.117)	(0.106)
0.055	0.085	-0.076	0.252	-0.208	0.091	-0.085	0.248	-0.223
	(0.053)	(0.054)	(0.118)	(0.103)	(0.056)	(0.06)	(0.118)	(0.109)
0.060	0.088	-0.077	0.258	-0.21	0.095	-0.09	0.255	-0.231
	(0.054)	(0.055)	(0.119)	(0.103)	(0.059)	(0.063)	(0.119)	(0.114)
0.065	0.091	-0.079	0.263	-0.213	0.100	-0.095	0.262	-0.239
	(0.055)	(0.055)	(0.12)	(0.103)	(0.062)	(0.068)	(0.121)	(0.12)
0.070	0.093	-0.08	0.269	-0.216	0.105	-0.099	0.27	-0.247
	(0.056)	(0.056)	(0.121)	(0.103)	(0.066)	(0.072)	(0.122)	(0.126)
0.075	0.096	-0.082	0.275	-0.218	0.11	-0.104	0.277	-0.255
	(0.058)	(0.056)	(0.122)	(0.102)	(0.069)	(0.077)	(0.124)	(0.133)
0.080	0.099	-0.083	0.281	-0.221	0.115	-0.109	0.284	-0.263
	(0.059)	(0.056)	(0.123)	(0.102)	(0.074)	(0.082)	(0.125)	(0.14)
0.085	0.102	-0.085	0.287	-0.223	0.121	-0.114	0.291	-0.271
	(0.06)	(0.057)	(0.124)	(0.102)	(0.078)	(0.087)	(0.127)	(0.147)
0.090	0.104	-0.086	0.292	-0.226	0.126	-0.119	0.299	-0.279
	(0.061)	(0.057)	(0.124)	(0.102)	(0.083)	(0.093)	(0.129)	(0.154)
0.095	0.107	-0.088	0.298	-0.228	0.132	-0.124	0.306	-0.286
	(0.062)	(0.057)	(0.125)	(0.101)	(0.088)	(0.098)	(0.13)	(0.162)
0.100	0.11	-0.089	0.304	-0.23	0.138	-0.129	0.313	-0.294
	(0.063)	(0.058)	(0.126)	(0.101)	(0.093)	(0.104)	(0.132)	(0.169)
0.105	0.113	-0.09	0.31	-0.232	0.144	-0.134	0.32	-0.301
	(0.064)	(0.058)	(0.127)	(0.1)	(0.099)	(0.11)	(0.134)	(0.177)
0.110	0.116	-0.092	0.315	-0.234	0.15	-0.139	0.326	-0.308
	(0.065)	(0.058)	(0.127)	(0.1)	(0.105)	(0.116)	(0.135)	(0.184)
Slope [a]	0.517	-0.297	1.135	-0.551	0.924	-0.906	1.429	-1.615

Source: Understanding Society (2015), Waves 1-5, 2009-2014; linked with DfT Accessibility Statistics 2013. Standard errors in parenthesis. [a] Represents the rate of increase, in percentage points, of the APE when the local unemployment rate increases by 1 percentage point. *Example*: According to Model 1, if the local unemployment rate increases by 1percentage point, the APE of the short-term unemployed to become unemployed in the next period increases by 0.52 percentage points.
