# Supplementary material for: Local unemployment changes the springboard effect of low pay: Evidence from England
Source: PLoS One. 2019 Nov 13;14(11):e0224290. doi: 10.1371/journal.pone.0224290 (PMC6853294; doi:10.1371/journal.pone.0224290)
Supplement: S1 File — (PDF) [file pone.0224290.s009.pdf]

S1 File. Average Partial Effects

(1) Average partial effect of becoming unemployed for someone short-term unemployed (compared to someone low-paid employed)

$$\begin{aligned}\text{Partial effect}_i^{(Ue)} &= \Phi\left[\left(\hat{\gamma}_{13} + \text{ue-rate} \times \hat{\delta}_{13} + \bar{x}_{it}'\hat{\beta}_1 + y_{i0}^{(hp)}\hat{\tau}_{11} + y_{i0}^{(lp)}\hat{\tau}_{12} + y_{i0}^{(ue\text{-}short)}\hat{\tau}_{13} + \bar{x}_i\hat{\pi}_1 + z_i y_{i0}^{(hp)}\hat{\eta}_{11} + z_i y_{i0}^{(lp)}\hat{\eta}_{12} + z_i y_{i0}^{(ue\text{-}short)}\hat{\eta}_{13}\right)\left(\sqrt{1-\hat{\lambda}_1}\right)\right] \\ &- \Phi\left[\left(\hat{\gamma}_{12} + \text{ue-rate} \times \hat{\delta}_{13} + \bar{x}_{it}'\hat{\beta}_1 + y_{i0}^{(hp)}\hat{\tau}_{11} + y_{i0}^{(lp)}\hat{\tau}_{12} + y_{i0}^{(ue\text{-}short)}\hat{\tau}_{13} + \bar{x}_i\hat{\pi}_1 + z_i y_{i0}^{(hp)}\hat{\eta}_{11} + z_i y_{i0}^{(lp)}\hat{\eta}_{12} + z_i y_{i0}^{(ue\text{-}short)}\hat{\eta}_{13}\right)\left(\sqrt{1-\hat{\lambda}_1}\right)\right]\end{aligned} \quad (1)$$

With ue-rate $\in \{0.005, 0.010, 0.015, \ldots, 0.115\}$ and $APE^{(Ue)} = \frac{\sum_{i=1}^{N}\text{Partial effect}_i^{(Ue)}}{N}$.

(2) Average partial effect of becoming higher-paid employed for someone short-term unemployed (compared to someone low-paid employed)

$$\begin{aligned}\text{Partial effect}_i^{(Hp)} &= \Phi\left[-\left(\hat{\gamma}_{13} + \text{ue-rate} \times \hat{\delta}_{13} + \bar{x}_{it}'\hat{\beta}_1 + y_{i0}^{(hp)}\hat{\tau}_{11} + y_{i0}^{(lp)}\hat{\tau}_{12} + y_{i0}^{(ue\text{-}short)}\hat{\tau}_{13} + \bar{x}_i\hat{\pi}_1 + z_i y_{i0}^{(hp)}\hat{\eta}_{11} + z_i y_{i0}^{(lp)}\hat{\eta}_{12} + z_i y_{i0}^{(ue\text{-}short)}\hat{\eta}_{13}\right)\left(\sqrt{1-\hat{\lambda}_1}\right)\right] \\ &\times \Phi\left[\left(\hat{\gamma}_{23} + \text{ue-rate} \times \hat{\delta}_{23} + \bar{x}_{it}'\hat{\beta}_2 + y_{i0}^{(hp)}\hat{\tau}_{21} + y_{i0}^{(lp)}\hat{\tau}_{22} + y_{i0}^{(ue\text{-}short)}\hat{\tau}_{23} + \bar{x}_i\hat{\pi}_2 + z_i y_{i0}^{(hp)}\hat{\eta}_{21} + z_i y_{i0}^{(lp)}\hat{\eta}_{22} + z_i y_{i0}^{(ue\text{-}short)}\hat{\eta}_{23}\right)\left(\sqrt{1-\hat{\lambda}_2}\right)\right] \\ &- \Phi\left[-\left(\hat{\gamma}_{12} + \text{ue-rate} \times \hat{\delta}_{13} + \bar{x}_{it}'\hat{\beta}_1 + y_{i0}^{(hp)}\hat{\tau}_{11} + y_{i0}^{(lp)}\hat{\tau}_{12} + y_{i0}^{(ue\text{-}short)}\hat{\tau}_{13} + \bar{x}_i\hat{\pi}_1 + z_i y_{i0}^{(hp)}\hat{\eta}_{11} + z_i y_{i0}^{(lp)}\hat{\eta}_{12} + z_i y_{i0}^{(ue\text{-}short)}\hat{\eta}_{13}\right)\left(\sqrt{1-\hat{\lambda}_1}\right)\right] \\ &\times \Phi\left[\left(\hat{\gamma}_{22} + \text{ue-rate} \times \hat{\delta}_{23} + \bar{x}_{it}'\hat{\beta}_2 + y_{i0}^{(hp)}\hat{\tau}_{21} + y_{i0}^{(lp)}\hat{\tau}_{22} + y_{i0}^{(ue\text{-}short)}\hat{\tau}_{23} + \bar{x}_i\hat{\pi}_2 + z_i y_{i0}^{(hp)}\hat{\eta}_{21} + z_i y_{i0}^{(lp)}\hat{\eta}_{22} + z_i y_{i0}^{(ue\text{-}short)}\hat{\eta}_{23}\right)\left(\sqrt{1-\hat{\lambda}_2}\right)\right]\end{aligned} \quad (2)$$

With ue-rate $\in \{0.005, 0.010, 0.015, \ldots, 0.115\}$ and $APE^{(Hp)} = \frac{\sum_{i=1}^{N}\text{Partial effect}_i^{(Hp)}}{N}$. As the composite error term is not standard-normal distributed $\left(\sigma_{v_j}^2 \neq 1 \text{ with } j \in \{1,2\}\right)$ we follow the suggestion of Arulampalam (1999) to correct the coefficients.
